# The information content of ultrafast observables: spectroscopy and scattering in excited norbornadiene

**DOI:** 10.1039/d6fd00085a

**Published:** 2026-11-11

**Authors:** J. C. Cooper, A. Kirrander

**Affiliations:** a Physical and Theoretical Chemistry Laboratory South Parks Road Oxford OX1 3QZ UK adam.kirrander@chem.ox.ac.uk +44 (0)1865 275422

## Abstract

We assess the sensitivity of time-resolved observables for characterising ultrafast nonadiabatic reaction dynamics. Using the ultrafast (<100 fs) photoisomerisation of norbornadiene as a testbed, we compare the observables for trajectory surface hopping simulations carried out at four different levels of electronic structure theory, SA-CASSCF and XMS-CASPT2 with (2,2) and (4,4) active spaces, resulting in four different sets of dynamics with distinct lifetimes and branching ratios. For each simulation, we calculate time-resolved photoelectron spectra, and electron- and X-ray scattering signals. The observables are critically evaluated and the advantages and disadvantages of each observable for characterising the excited state dynamics are discussed, including the level of theory required to accurately predict the observables and links to recent and future experiments. Broadly, photoelectron spectroscopy tracks electronic character and internal conversion, while X-ray and electron scattering resolve structural dynamics with the potential for additional information relating to electronic structure. We conclude that multimodal detection is required to uniquely constrain the underlying molecular dynamics.

## Abstract 2

How sensitive are different observables for characterising ultrafast nonadiabatic dynamics? We assess this using four different simulations and three observables in the molecular photoswitch norbornadiene.

## Graphical abstract

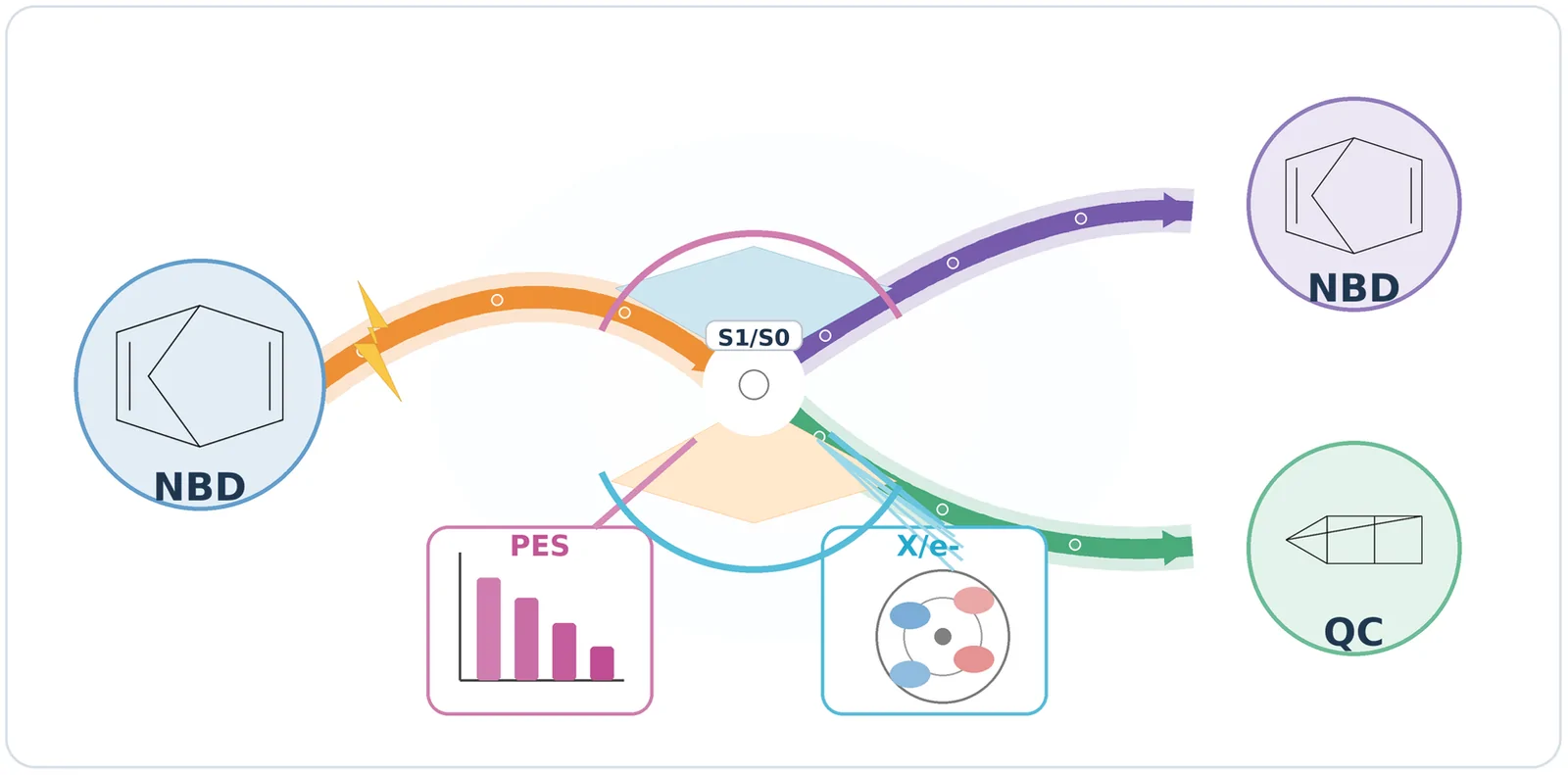

## 1 Introduction

Excited-state dynamics constitutes a central challenge in chemical physics.^1^ It sits at the intersection of electronic structure, nuclear dynamics, and experiments on ultrafast timescales. The electronic structure for excited states may involve a strong multiconfigurational character, intermixed valence and Rydberg manifolds, significant contributions from doubly excited configurations, and, at sufficiently high energy, coupling to ionisation continua.^2–4^ These electronic complexities are inseparable from the nuclear dynamics; photoexcitation launches coupled electron–nuclear wavepackets on potential energy surfaces that may intersect or mix through nonadiabatic couplings. Quantitative predictions require accurate excited-state electronic structure and a treatment of the dynamics capable of resolving competing relaxation and decay mechanisms, including internal conversion, intersystem crossing, predissociation, fragmentation, and possibly even autoionisation.^4–10^ The resulting processes often occur on femtosecond timescales and generate transient species that challenge both experiment and computations. Consequently, high-level electronic-structure theory, nonadiabatic nuclear dynamics, and time-resolved measurements are essential for understanding photochemical reactivity, radiation-induced processes, and, more broadly, the flow of energy and charge in molecular systems.

Experiments have the advantage that they measure the true dynamics, bar systematic or accidental errors and unavoidable limitations on resolution and statistics. Significant efforts have been made to develop inversion methods that translate experimental data into a molecular model, especially in the context of scattering experiments.^11–23^ Generally, the inverse problem is ill-posed and underdetermined, suffering from instability and non-uniqueness.^24–26^ Given that experiments project complex multidimensional dynamics onto lower-dimensional observables, information loss is unavoidable. A possible solution is to carry out multimodal measurements and combine complementary data.^27^ However, given the technical challenges associated with any ultrafast measurement and the difficulty in reproducing conditions across experiments, this is nontrivial. Instead, a common strategy is to present experimental results alongside supporting theory and simulations to aid interpretation and analysis.^28–41^ Published results generally demonstrate close agreement between theoretical predictions and experimental data, often without systematic consideration of confirmation bias.

This is particularly striking given that the accuracy and convergence of excited-state electronic structure and nonadiabatic dynamics is strongly dependent on methodology and execution. Benchmarks against higher-level theory are possible, at least in model systems, but comparison to experimental data is an essential part of the validation process. This intertwined relationship between theory and experiment is associated with potential pitfalls. Responses to the situation include ongoing theoretical method development and innovation,^42–44^ a closer scrutiny of the sensitivity of simulations with respect to electronic structure calculations and simulation parameters,^45–49^ benchmarks in models where theoretically correct answers are known,^50–53^ and establishment of best practice guidelines.^54^ An interesting take on the issue is the recent *Cyclobutanone Challenge*, which invited researchers to publish electronic scattering predictions without prior knowledge of the experiment.^55–69^

The goal of this paper is to explore whether agreement between theory and experiment necessarily implies that the theoretical dynamics is correct, and the degree to which different experiments can discriminate between theoretical models. To address this question, we exploit four distinct nonadiabatic excited state simulations of the same molecule. For each of the simulations, we calculate several different experimental observables, comparing complete theoretical predictions of both the dynamics and the observables. This enables us to see which observable, or combination of observables, can best distinguish between the models and determine the features in the dynamics that each observable is most sensitive to. Equally, one can consider each simulation an “alternate reality” and examine how different the experimental results would be for each of these realities. In terms of the observables, our main focus is on X-ray scattering and photoelectron spectroscopy, however, we also consider ultrafast electron diffraction. Scattering and spectroscopy have a natural complementarity, with scattering effectively tracking structural dynamics and spectroscopy being more sensitive to electronic changes in the target.^27,40,70,71^

The specific system we examine is the extensively studied ultrafast norbornadiene (NBD) ↔ quadricyclane (QC) photoswitch.^40,71–78^ In addition to the fundamental photochemical interest of the reaction, this photoswitch isomerisation is exploited in solar energy harvesting applications.^79,80^ In the following, we exploit previous work on the electronic structure and the dynamics.^49,81^ The simulations we use are based on four different levels of electronic structure theory; SA(3)-CASSCF(2,2) and (4,4), accompanied by the corresponding XMS-CASPT2 level of calculations.^81^ For each model of the electronic structure, fewest-switches surface hopping simulations exhibit significant qualitative differences, *e.g.* SA(3)-CASSCF(2,2) *vs.* SA(3)-CASSCF(4,4), but also more subtle differences, *e.g.* SA(3)-CASSCF(2,2) *vs.* XMS-CASPT2(4,4).^49^ Herein, we present the dynamics and observables for the different models, and discuss the limitations of each observable as well as their corresponding complementarity.

## 2 Theory

### 2.1 Simulations

In the following, we consider simulations carried out using mixed-quantum classical (MQC) nonadiabatic dynamics. This approximates the time-evolution of the system as a sum over *N*_trj_ classical point trajectories,1
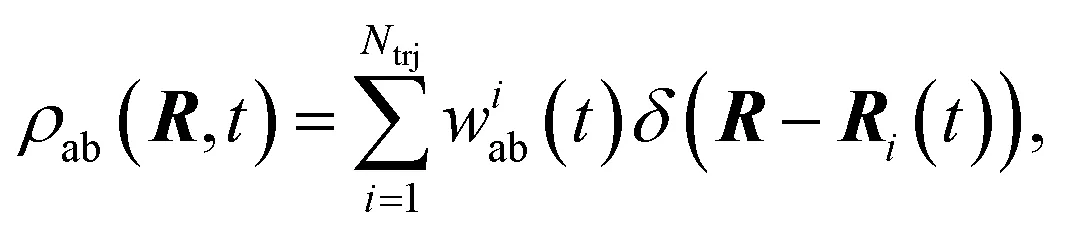
where *ρ*_ab_(***R***, *t*) is the electronic density element at nuclear geometry ***R***, *t* is the time, and *δ*(***R*** − ***R***_*i*_(*t*)) the Dirac delta function. Each trajectory is defined by its sequence of nuclear geometries, {***R***_*i*_(*t*)}, and the weight of each element of the electronic density, *w*^*i*^_ab_(*t*) for electronic states a and b.

Different MQC methods will provide different prescriptions for the propagation of the two variables; here, we employ Tully’s fewest switches surfaces hopping (FSSH). This localises each trajectory’s electronic density to a single adiabatic state, given by the index *λ*_*i*_(*t*) denoted the active state,2
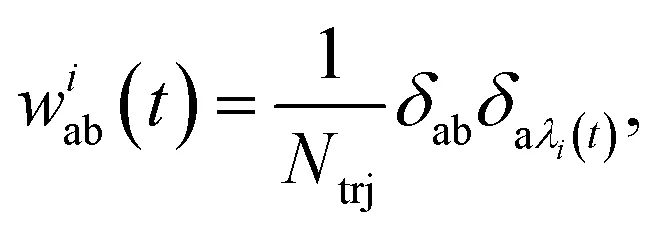
where *N*_trj_^−1^ is a normalisation factor that weights each trajectory equally and *δ*_*ij*_ is the Kronecker delta. The coordinate vector ***R***_*i*_(*t*) is propagated classically on the active state, which can change during the dynamics. Further details on the evolution of these quantities are provided elsewhere.^9,82^

In practice, a large number of trajectories *N*_trj_ are propagated.^45^ The key benefit of MQC dynamics methods is their compatibility with modern *ab initio* electronic structure methods,^47,54^ which provide the potential energy surfaces (and non-adiabatic couplings) for the propagation. The initial conditions are obtained by Wigner sampling,^47,54^ assuming instantaneous vertical excitation at *t* = 0. The energy bandwidth of the excitation pulse is taken into account in the initial sampling,^54,83^ and the temporal duration of the excitation pulse (pump) is then included phenomenologically *via* a convolution (see Section 2.4).

The prediction of experimental observables is essential given our focus here on time-resolved pump–probe experiments, where the observable is given as a function of the time delay *t* between the initial excitation (pump) and the measurement (probe). For trajectory simulations, we use the formula3
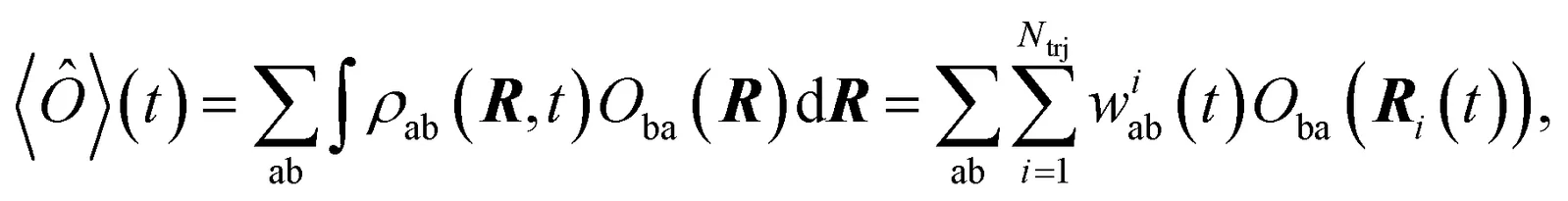
where *Ô* is the operator for the observable and *O*_ba_(***R***) is its position- and electronic-density-element-dependent form. For FSSH, eqn (2) simplifies this to,4
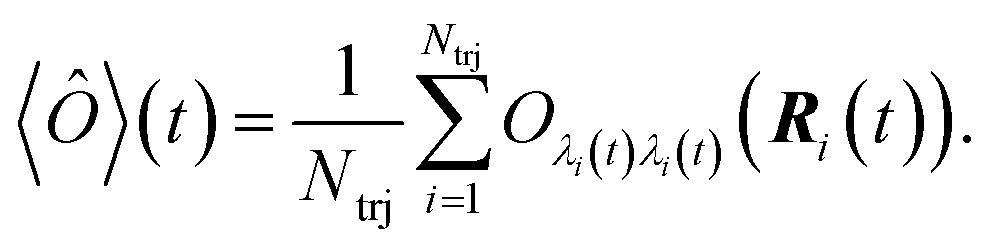


In short, the ensemble average of each observable is calculated, with the only contribution coming from the active states.

### 2.2 X-ray and electron scattering

In a gas-phase X-ray scattering experiment, an ensemble of isolated molecules is intersected by an incident X-ray pulse with wave vector ***k***_0_, and photons scattered into wave vector ***k***_s_ are recorded on a two-dimensional detector.^84,85^ The momentum transfer is

5 ***q*** = ***k***_0_ − ***k***_s_, *q* = |***q***|.

For randomly oriented molecules, or for the isotropic component of an aligned distribution, the measured signal is rotationally averaged and depends only on *q*.^86^ Since gas-phase detectors are typically not energy resolving, the measured observable is usually the total, energy-integrated scattering signal rather than separated elastic or inelastic contributions.

The nonresonant X-ray scattering cross section can be written as6
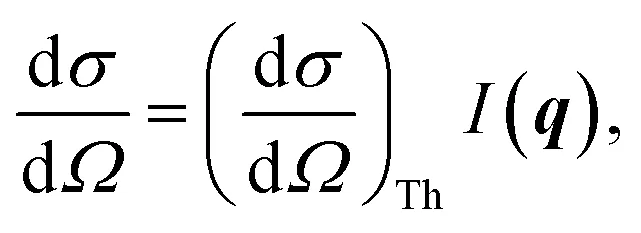
where (d*σ*/d*Ω*)_Th_ = *r*_0_^2^***K̂*** is the Thomson cross section with *r*_0_ the classical electron radius and ***K̂*** the polarisation dependence, and *I*(***q***) is the molecular scattering factor. This is calculated using the scattering operator 
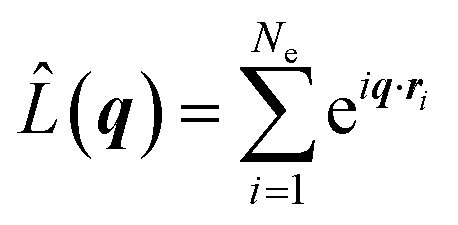
, where *N*_e_ is the number of electrons and ***r***_*i*_ are the coordinates of electron *i*. For a molecule with nuclear geometry ***R*** initially in electronic state a, the state-resolved scattering into a final electronic state b is7
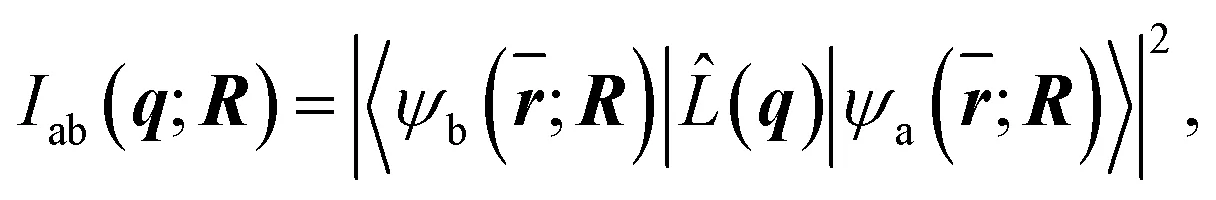
where *ψ*_a_(***r̄***; ***R***) is the electronic wavefunction for state *a*, which is a function of the electronic coordinates ***r̄*** and has parametric dependence on the nuclear coordinates ***R***. In our case, we shall only look at the rotationally averaged signal, which gives the isotropic portion of the signal on the detector, giving8
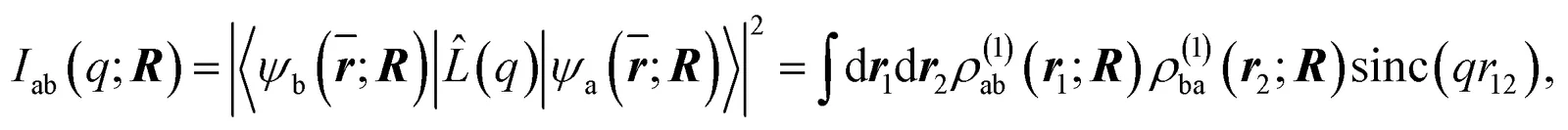
where *r*_12_ = |***r***_1_ − ***r***_2_| is the inter-electronic separation and *ρ*^(1)^_ab_(***r***; ***R***) the one-electron reduced (transition) density for states a and b at nuclear geometry ***R***. The sinc-function has the standard definition sinc(*x*) = sin(*x*)/*x*. When *a* = *b*, the expression in eqn (8) returns the elastic term, and *a* ≠ *b* returns an inelastic term.

The total energy-integrated signal can be shown to be^84,87,88^9
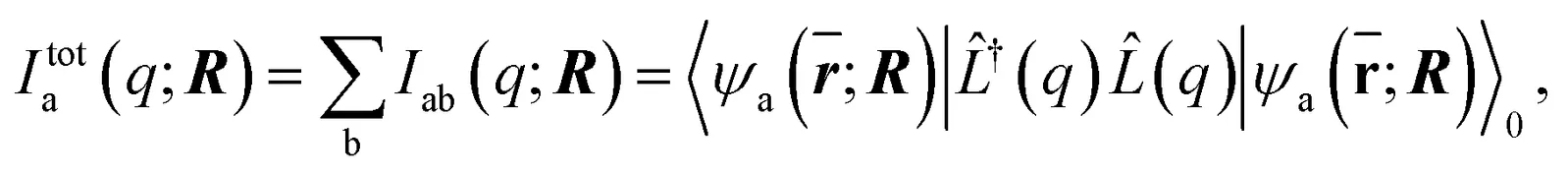
where the subscript zero implies rotational averaging over molecular orientations, which means that the scattering becomes a function of the momentum transfer *q* (rather than the vector ***q***). This expression can be rewritten in terms of the two-electron reduced density *ρ*^(2)^_aa_(***r***_1_, ***r***_2_; ***R***) as10
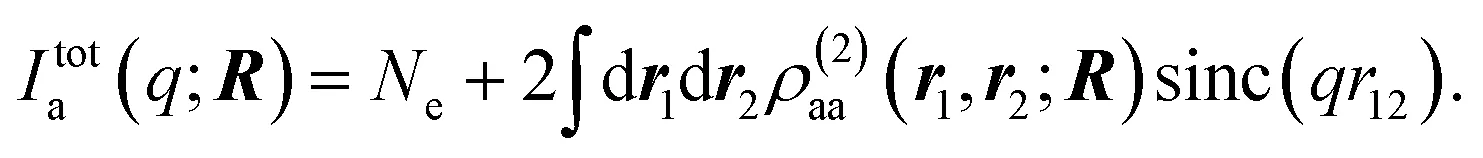


The elastic, inelastic, and total scattering defined above can be calculated directly from electronic wavefunctions obtained from *ab initio* electronic structure methods.^87–92^ In practice, however, many use a simpler approximation. The independent atom model (IAM) assumes that the molecular electron density is a sum of non-interacting, spherically symmetric atomic densities centred at the nuclear positions. For an isotropic ensemble of molecules with atoms m and n at positions ***R***_m_ and ***R***_n_, with the interatomic distance *R*_mn_ = |***R***_m_ − ***R***_n_|, the IAM elastic intensity is given by the expression11
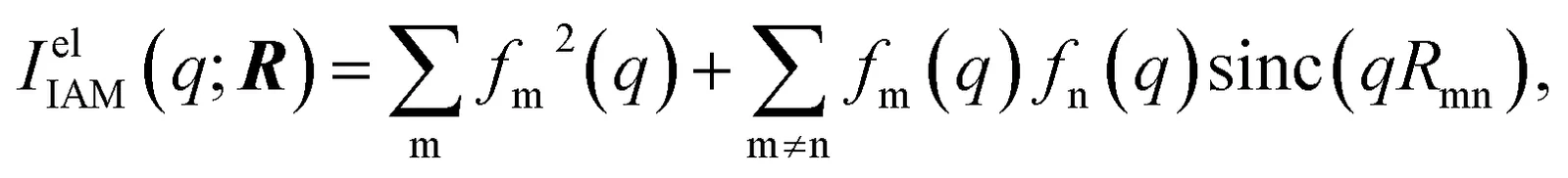
where *f*_m_(*q*) is the X-ray atomic form factor for atom m.^93^ The first term is a constant atomic contribution, while the second term contains the structural information *via* the interatomic distances. The IAM inelastic contribution is usually included as an incoherent sum of tabulated atomic Compton scattering factors,12
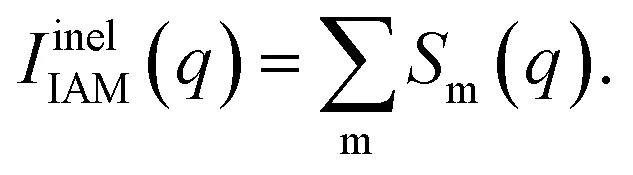
This does not depend on the nuclear geometry, so the total scattering in IAM is given by13
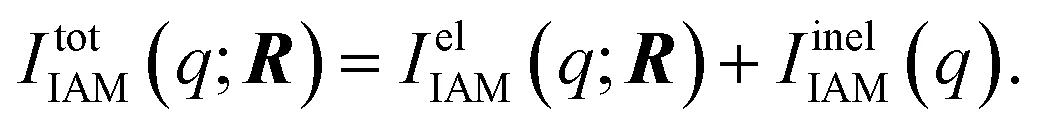


The IAM captures the dominant dependence of the signal on molecular geometry, but omits changes in scattering due to chemical bonding, correlation, and the character of the electronic state.^89,90,94^ This limitation is especially relevant in photoexcited dynamics, where both the nuclear geometry and the electronic density can change significantly following excitation.^33,95–97^

For a trajectory ensemble, the scattering signal can be evaluated by inserting the state- and geometry-dependent scattering observable into eqn (4),14
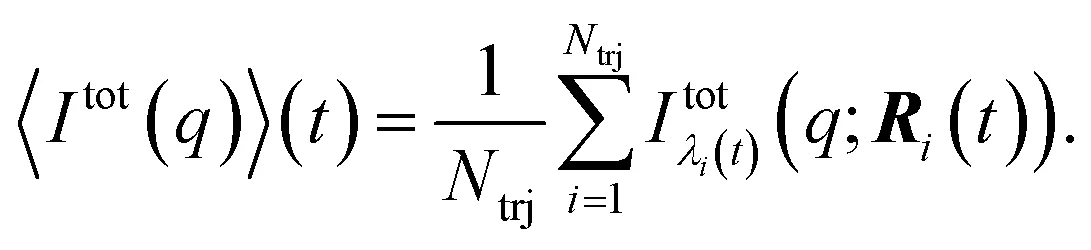


Later, we will refer to the observable calculated in eqn (14) as *I*(*t*, *q*) (see *e.g.*eqn (31)). Within the IAM, the scattering is independent of the electronic state, so the state label can be omitted.

#### 2.2.1 Electron scattering

Electron scattering can be formulated similarly to X-ray scattering.^84,98,99^ First, we replace the Thomson cross section with the Rutherford cross section, 4/(*a*_0_^2^*s*^4^), where *a*_0_ is the Bohr radius, and, by convention, the momentum transfer is denoted *s* rather than *q*.

The rotationally averaged signal can then be written as a sum over electron–electron, electron–nuclear, and nuclear–nuclear terms,^84^

15 *I*^e,tot^_a_(*s*;***R***) = 4/(*a*_0_^2^*s*^4^)[*I*^tot^_ee_(*s*;***R***) − 2*I*_eN_(*s*;***R***) + *I*_NN_(*s*;***R***)]

16
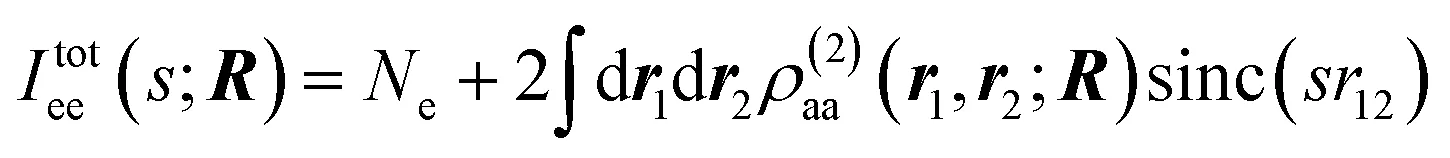
17
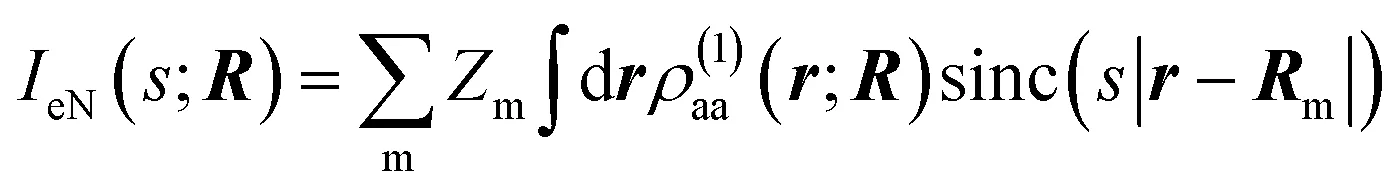
18
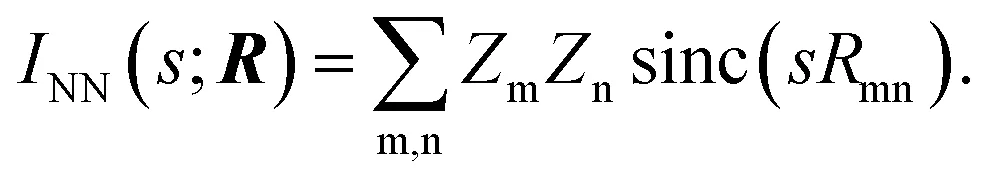


This is the *ab initio* analogue of the X-ray expression, but with the important addition that the incoming electron scatters from both the molecular electrons and the nuclei, *i.e.* the charge density of the system including the charge on each of the nuclei, *Z*_m_. We note that the electron–electron term is identical to the X-ray scattering term of eqn (10). For the IAM, it suffices to use electron form factors, calculated through the Mott–Bethe relation,^98,99^19
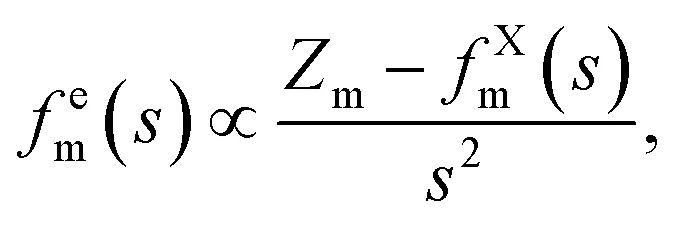
and eqn (11) and (13).‡‡The proportionality constant 2*m*_e_e^2^/*ħ*^2^, where *m*_e_ is the mass and *e* the charge of an electron, turns eqn (19) into an equality. Thus, the IAM electron-scattering signal retains the same structural interpretation as the X-ray IAM signal, but the atomic factors include the effect of scattering from the nuclear charge. The resulting trajectory-averaged electron-scattering observable is constructed the same way as for X-rays, *via*eqn (14).

### 2.3 Photoelectron spectroscopy

In valence photoelectron spectroscopy, a gas-phase ensemble of isolated molecules is ionised by an ultraviolet pulse with central photon energy *ℏω*_0_. The absorption of one photon ejects an electron into the continuum and leaves the molecule in a cationic electronic state, with the experiment measuring the kinetic-energy distribution of the outgoing electrons.§§The angular distribution of the photoelectrons is also able to be determined, but we do not consider it here. For ionisation from neutral electronic state a to cationic state b, the vertical binding energy at the nuclear geometry ***R*** is20
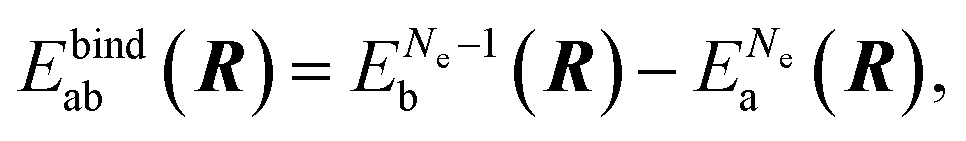
so the photoelectron kinetic energy is determined by energy conservation,

21 *E*^ab^_kin_(***R***;*ω*_0_) = *ℏω*_0_ − *E*^bind^_ab_(***R***).

The effect of the bandwidth of the ultraviolet pulse is discussed in Section 2.4 below.

Within the dipole approximation, the channel-resolved photoionisation amplitude is a transition dipole moment between the initial neutral state and the final continuum state including the outgoing electron *ψ*^(–)^_***k***_(***r***) and the cation 
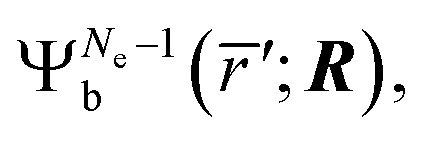
22
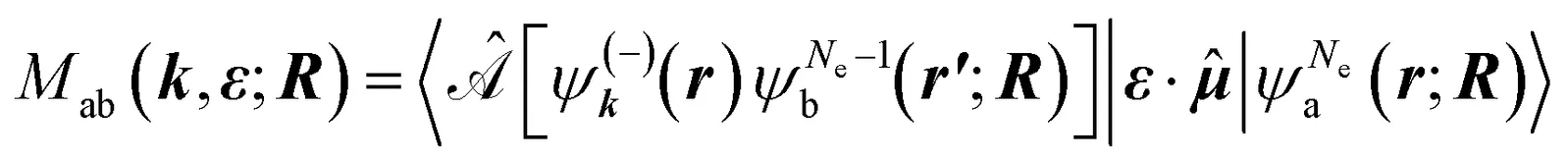
Here, ***r*** is the coordinate of a single electron, *r̄*′ the coordinates of *N*_e_ − 1 electrons, ***ε*** the polarisation vector of the ionising (probe) pulse, ***μ̂*** the molecular dipole operator, and the operator 
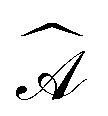
 enforces antisymmetry. In the continuum wavefunction *ψ*^(−)^_**k**_ the subscript ***k*** gives the momentum of the outgoing electron, 
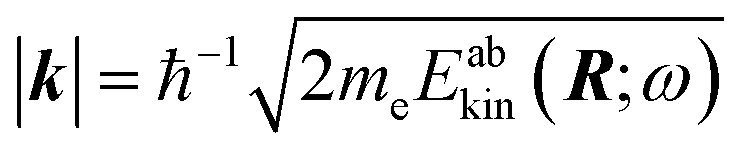
. For the rotationally averaged gas-phase samples we consider, the corresponding cross section is then *σ*_ab_(*ω*; ***R***) = *C*(*ω*, *k*_ab_)|*M*_ab_(***k***_ab_, ***ε***;***R***)|^2^ where *C*(*ω*, *k*_ab_) contains the photon-energy prefactor, the density of continuum states, the polarisation factor, and fundamental constants. A fixed-geometry photoelectron spectrum can be written as a sum over cationic final states,23
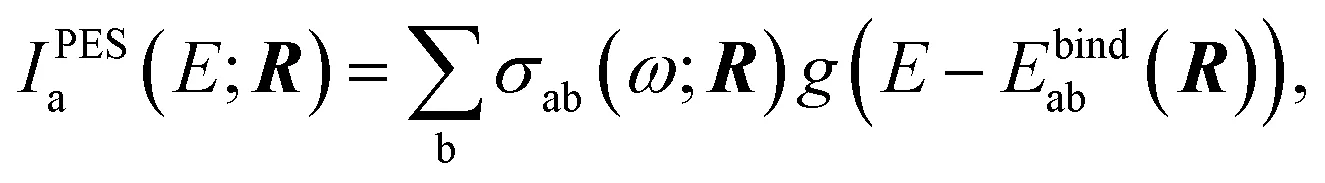
where *g*(*E*) is a lineshape function representing the experimental energy resolution. We choose a constant width Gaussian (see Section 2.4 below for further details) as a simple approximation, but more sophisticated methods exist to inform this choice.^100,101^

In practice, calculating the full photoionisation cross section is challenging due to interaction with the outgoing electron.^102–107^ A common approximation is therefore to use Dyson orbitals, *φ*_ab_(***R***), the overlap between the neutral *N*_e_-electron wavefunction and the cationic (*N*_e_ − 1)-electron wavefunction at nuclear geometry ***R***.^104^ The intensity of an ionisation channel can be approximated by the norm, 
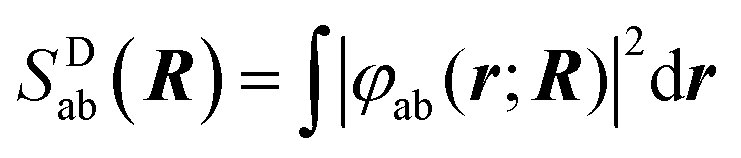
, giving24
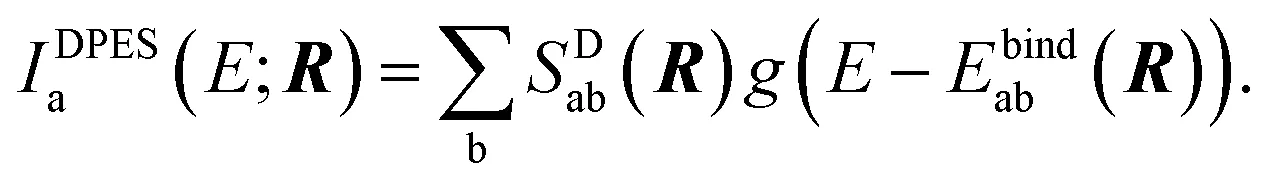


This approximation neglects the photon-energy, angular, and continuum-scattering dependence of the full transition dipole matrix element, and is therefore only useful for assigning bands and estimating relative channel strengths. As with scattering, the trajectory-averaged signal is obtained by inserting the cross-section into eqn (4), 25
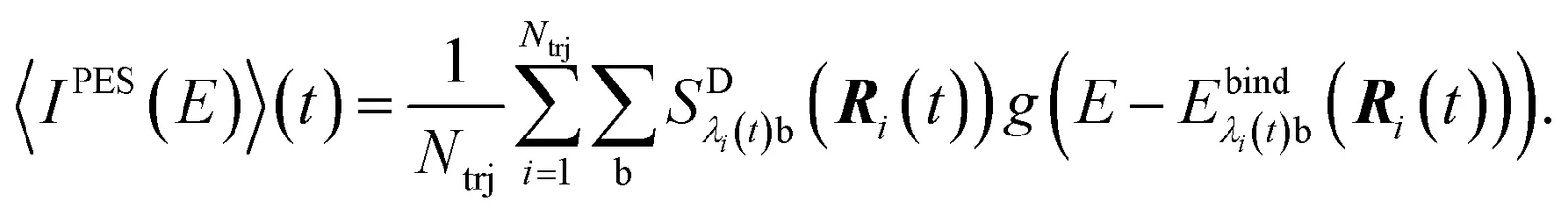


Again, we have a series of observables, this time parametrised by the measured binding energy *E*. Later, we will refer to the quantity calculated in eqn (25) as *σ*_PES_(*t*, *E*_BE_).

### 2.4 Experimental resolution

For Gaussian pulses, the time-bandwidth product *P*^tb^ is given by

26 *P*^tb^ = Δ*E*Δ*t* ≥ 4*ℏ*ln2

where Δ*E* and Δ*t* are the full-widths-at-half-maximum (FWHMs) in energy and time respectively. In ref. 49, we used a top-hat excitation window between 5.8 and 6.2 eV. For simplicity, we use a Gaussian function of equivalent variance in the following, meaning that the FWHM is 
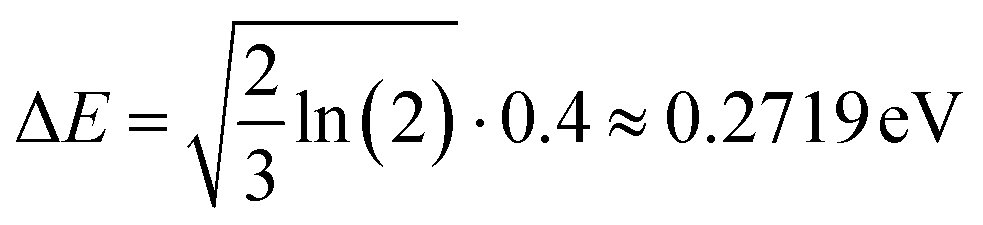
. Henceforth, we shall assume that all pulses are Gaussian, and all pulses are Fourier-limited (*i.e.*, that they satisfy the equality in eqn (26)). For the excitation pulse studied, this gives a FWHM in time of Δ*t*_pu_ ≈ 6.71 fs, where the subscript ‘pu’ indicates this is the pump pulse. We cannot change the pulse *ex post facto*, and thus this represents an inherent limit on the time resolution for our dynamics.

On the other hand, the probe pulse (subscript ‘pr’) can be adjusted post-simulation. The overall time uncertainty of the experimental setup is the cross-correlation of the pump and probe pulses, which gives a Gaussian width27
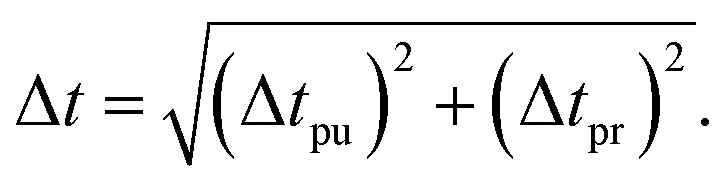
This value is the minimal time-uncertainty of the entire measurement process, and this is the temporal broadening applied to predicted observables. We use a convolution, defined as28
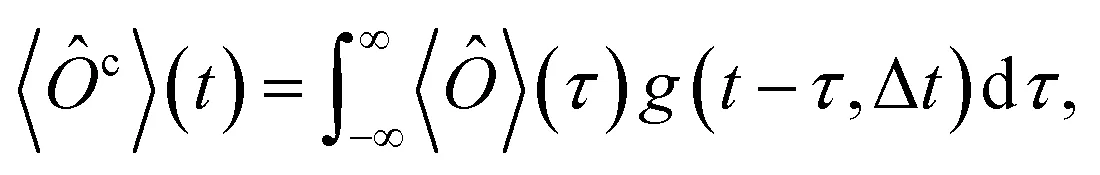
where the *c* denotes the convoluted observable, and *g*(*t*, Δ*t*) a normalised Gaussian with FWHM Δ*t*.

The energy bandwidth of the pump pulse is already accounted for in the selection of the initial conditions for the dynamics and does not require further consideration. Thus, the energy broadening applied to the measured observable is simply the bandwidth of the probe pulse, *i.e*. Δ*E* = Δ*E*_pr_. For the X-ray scattering, the bandwidth of the probe has negligible effect, since most scattering experiments use photon energies around 10 keV, and even for a 1 fs pulse duration the ratio Δ*q*/*q* ≈ 10^−4^ is small (likely smaller than the effect of the Waller–Hartree approximation itself).^84,93^ The scattering pattern is therefore calculated without broadening, using eqn (14). In time-resolved photoelectron spectroscopy, the picture is more involved. From eqn (21), the spread in the measured binding energy *E*^bind^_ab_ is given by29
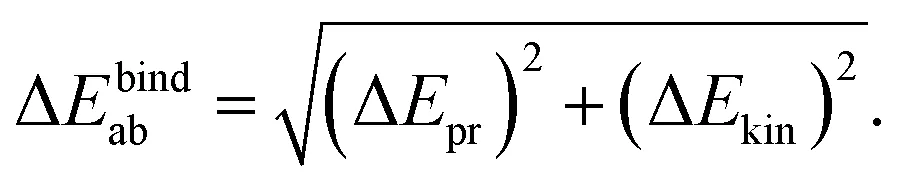


Assuming that the uncertainty in the measurement of the photoelectron kinetic energy is negligible (*i.e*. Δ*E*_kin_ ≈ 0), the uncertainty in the binding energy simply reflects the bandwidth of the probe pulse. Therefore, the photoelectron spectrum is broadened by setting the lineshape function in eqn (25) to30
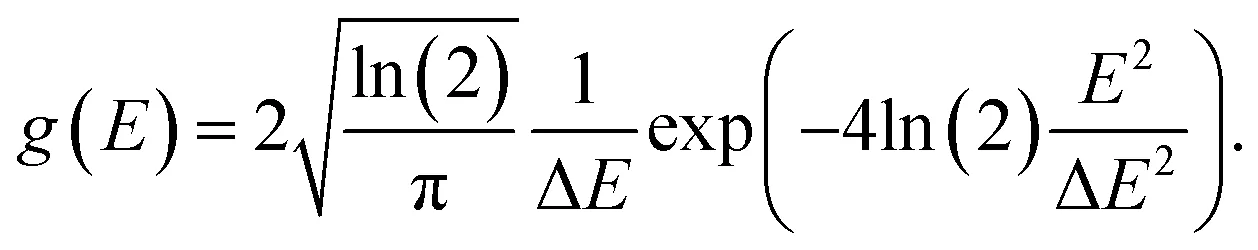


### 2.5 Previous work on NBD

In this work, we consider the dynamics of NBD after excitation to the low-lying S_1_ valence state, a classical example of a [2 + 2]-cycloaddition. The transition is symmetry forbidden but gains oscillator strength *via* Herzberg–Teller coupling. The excited molecules decay to the electronic ground state through a rhombic conical intersection.^49^ Characteristic molecular geometries, corresponding to the ground-state minima of NBD and QC, the S_1_/S_0_ minimum energy conical intersection (MECI), and an S_1_ potential minimum are shown in Fig. 1A.

**Fig. 1:**
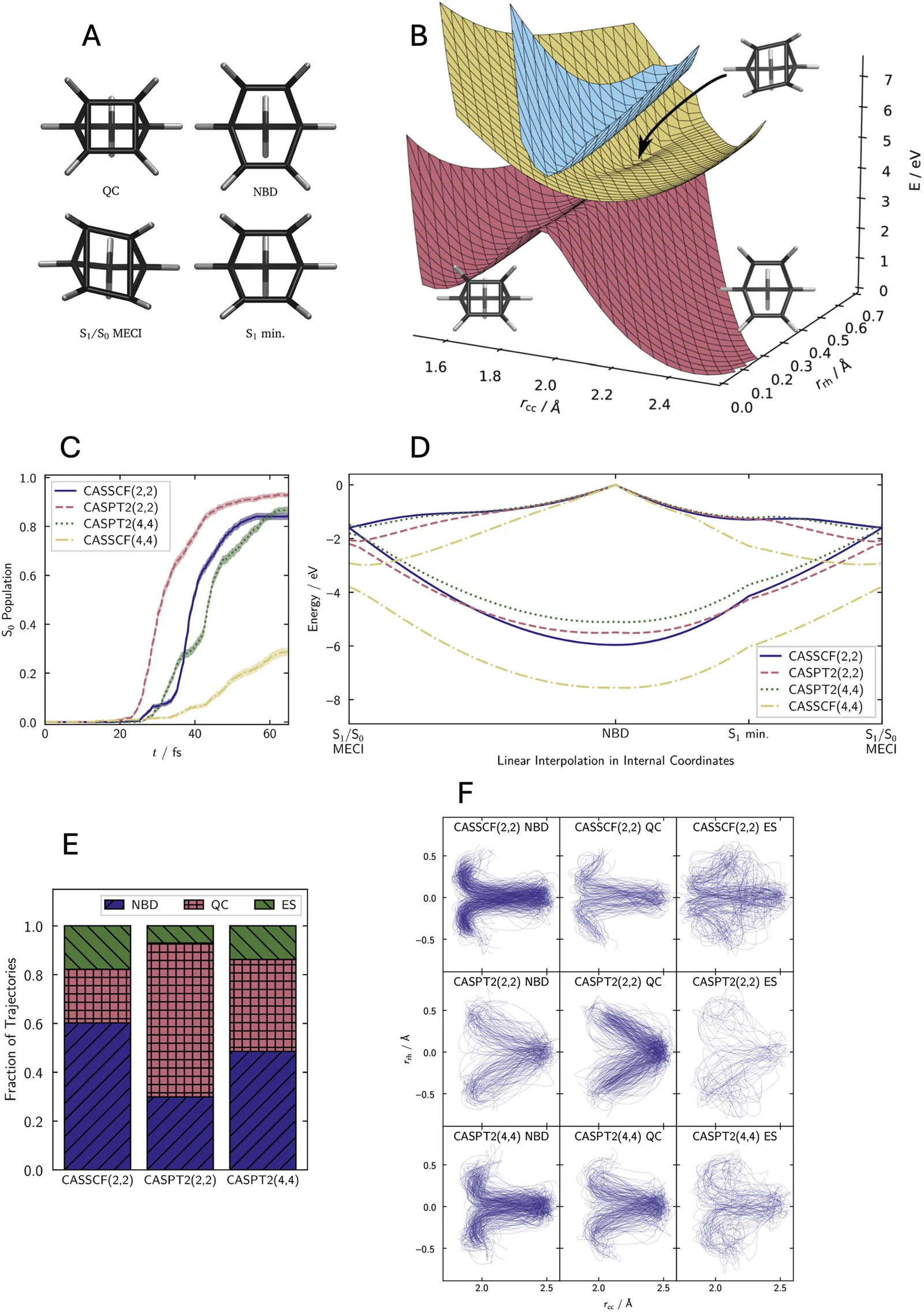
Overview of norbornadiene (NBD) and quadricyclane (QC). (A) QC and NBD ground state equilibrium geometries, minimum energy conical intersection S_1_/S_0_, and S_1_ minimum. (B) Schematic representation of S_0_, S_1_, and S2 potential energy surfaces as a function of the wing-separation coordinate *r*_cc_ and rhombicisation coordinate *r*_rh_. (C) S_0_ population. (D) Potential energy curves along a linear interpolation in internal coordinates (LIIC) from NBD to the MECI, both directly and *via* the S_1_ minimum. All calculations were performed on geometries optimised at the CASSCF(2,2) level. (E) Branching ratios at the end of the simulations for the similar first three models. (F) Trajectories grouped according to final product for the similar first three models. ((A) and (B) reproduced from ref. 81 with permission from Royal Society of Chemistry, copyright 2025. (C)–(F) reproduced from ref. 49 with permission from American Institute of Physics, copyright 2025).

In Fig. 1B, we show a reference potential energy surface as a function of two key coordinates, the wing-separation *r*_cc_ and the rhombicity *r*_rh_ (see ref. 49 for definitions). The former describes the motion between NBD and QC, while the latter describes the motion towards the MECI. As can be seen, the first excited state provides a smooth decay to the CI, which is situated on top of the ground state barrier. Thus, either minimum can be accessed from the MECI, primarily depending on the angle of approach to the MECI.^49^

The photoexcited dynamics of NBD upon UV excitation to the S_1_ state is represented by four different simulations, each yielding a different set of trajectories that are discussed in detail in ref. 49. The four simulations are run using identical initial conditions and propagation parameters, but different models for the electronic structure. Unsurprisingly, this has a strong impact on the simulation results. The four models are SA(3)-CASSCF(2,2), XMS-CASPT2(2,2), XMS-CASPT2(4,4), and SA(3)-CASSCF(4,4) (see potential energy curves in Fig. 1D, and further computational details in Section 3). The first three models give qualitatively similar results, while CASSCF(4,4) yields quite distinct outcomes, as evident from the population decays shown in Fig. 1C.

Starting first with the concordant simulations (*i.e.* excluding CASSCF(4,4)), the dynamics of NBD proceeds on the S_1_ state. This state is predominantly of HOMO → LUMO character, and quickly causes the two ‘wings’ of the molecule to move closer together. For CASSCF(2,2) and CASPT2(4,4), the molecule does not significantly break *C*_2v_ symmetry until it reaches a local minimum on the S_1_ state. Here, symmetry reduces to the C_2_ subgroup, splitting to the rhombic conical intersection (CI) with the ground state. CASPT2(2,2), on the other hand, does not have this local S_1_ minimum, and thus immediately breaks the symmetry post excitation, then continuing to the same CI.

We can classify these trajectories into three channels (pathways). The reactive, QC-forming channel (labelled ‘QC’), decays quickly to the QC ground state. The non-reactive, NBD-forming channel (labelled ‘NBD’), also decays quickly but moves back to the NBD ground-state potential energy well. We define both these channels as having reacted in the initial decay through the conical intersection (≈60 fs).^49^ Finally, the excited state (labelled ‘ES’) channel collects all of the slower-reacting trajectories, which exhibit delayed passage through the CI and which exhibit non-concerted oscillations in the excited state. The three channels, for each of the three models, are shown in Fig. 1F as trajectory traces in the *r*_cc_ and *r*_rh_ coordinates.

The dynamics in the three models leads to fairly similar population decay, as seen in Fig. 1C, but the different manner in which the trajectories approach the MECI region changes the dynamics on the electronic ground state. The CASSCF(2,2) simulation predominantly returns to NBD, with a 3 : 1 NBD : QC yield ratio. In contrast, the CASPT2(2,2) predominantly isomerises, resulting in a flipped almost 1 : 3 yield, while CASPT2(4,4) splits the difference, with a rather even 5 : 4 ratio, slightly in favour of NBD formation. The fraction of trajectories in each category, for each model, is shown in Fig. 1E.

The final method is CASSCF(4,4), which we include as an example of different, incorrect dynamics. This results from an electronic structure model quite different from the three models discussed above. It erroneously places the S_1_ and S_2_ states close together in energy, due to differential correlation; the (4,4) active space includes less correlation for S_1_ than S_2_.^81^ This leads to rapid population transfer between the two states, and significant differences in the dynamics—most importantly, a slower decay to S_0_. This causes excited-state dynamics that lasts well over 100 fs, while the other methods are almost completely decayed to the ground state by 60 fs. The classification scheme discussed above is thus not appropriate, and we shall not analyse the observables for CASSCF(4,4) in terms of these channels.

## 3 Computational details

The observables are calculated using geometries and electronic wavefunctions from the four simulations discussed in Section 2.5 above (and further detailed in ref. 49). In short, these are full-dimensionality surface hopping calculations using the energy-based decoherence correction, with initial conditions chosen from an initial Wigner sampling. The calculations used the four electronic structure methods detailed above; namely, SA(3)-CASSCF(2,2) and SA(3)-CASSCF(4,4), and the respective XMS-CASPT2 variants.

The scattering cross-sections are calculated either with the independent atom model (IAM) or *ab initio*. For IAM, we use the form factors taken from the *International Tables of Crystallography*.^93^ These calculations are performed at every time step using a momentum transfer grid in *q* (equivalently, *s*) of *q* ∈ [0, 10] a.u., with a Δ*q* spacing of 0.05 a.u. (1 a.u. ≈ 1.89 Å^−1^). For the *t* < 0 ground state reference, see eqn (31), we use a ground-state Wigner-sampled ensemble consisting of 10^4^ molecular geometries, see ref. 49, calculated at the CASSCF(2,2)/p-cc-(p)VDZ level of theory. *Ab initio* scattering calculations were performed using our in-house code.^88,89,91,92,108^ These calculations use Cartesian basis functions, which we checked provide energies and wavefunctions similar to the spherical basis functions. The *ab initio* scattering calculations are far more computationally demanding than IAM, and are thus performed only every 100 a.u. (≈2.4 fs) on a momentum-transfer *q* grid half as dense as for the IAM calculations. To further reduce the computational time required, the *ab initio* scattering calculations are performed only for a representative 50% of the CASSCF(2,2) dynamics ensemble, using the CASSCF(2,2) electronic structure for the wavefunction.

Photoionisation energies are calculated by the Δ-method, *i.e*. calculating the absolute energy differences between the doublet cationic states and the neutral singlet states (see Eq. (20)). We use the corresponding ionic electronic structure for each simulation, obtained by removing one active-space electron, *i.e*. SA(2)-CASSCF(1,2) for the CASSCF(2,2) simulations and SA(4)-CASSCF(3,4) for CASSCF(4,4), and the XMS-CASPT2 variations thereof. The state-averaging is chosen to ensure that all described channels are seen. The CASSCF calculations use Molpro 2022.2,^109^ the XMS-CASPT2(1,2) calculations OpenMolcas v23.02,^110^ and the XMS-CASPT2(3,4) calculations Bagel^111^ (commit 800fbe2). IP-LR-CC3 calculations are performed using the eT package.^112^ The p-cc-(p)VDZ basis is used for all electronic structure calculations, with density-fitting (Molpro/Bagel) or Cholesky decomposition (OpenMolcas). Further details on the electronic structure theory can be found in ref. 81.

Photoelectron cross-sections are calculated using the Dyson norm method as discussed around eqn (25) above. For XMS-CASPT2, the norms are calculated using the rotated CASSCF states.^39,113^ The photoelectron cross sections are calculated every 20 a.u. (≈0.5 fs), concurrently with the trajectory propagation, which ensures that the same wavefunctions are used as in the dynamics. The neutral wavefunction is used as the initial guess for the ionic wavefunction.

## 4 Scattering

### 4.1 X-ray scattering

Scattering experiments are ubiquitously shown as a percent-difference, Δ*%I*(*t*, *q*), which is given by31
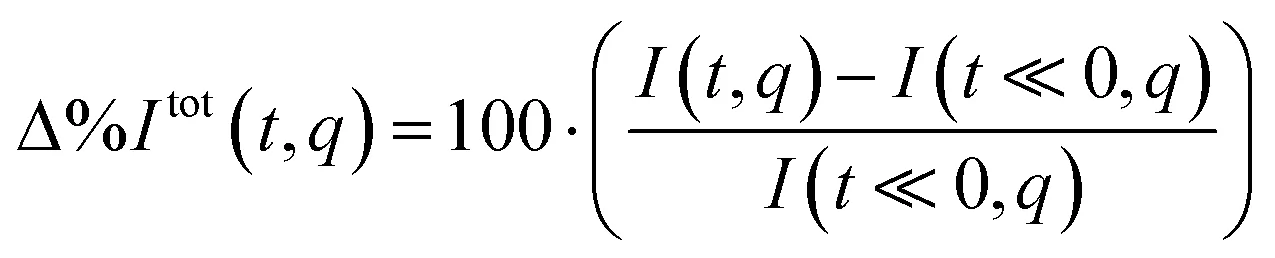
where *I*(*t*, *q*) is the scattering signal at pump-probe delay time *t* and momentum transfer *q* (see eqn (14)). For reference, in Fig. 2 we show the X-ray total percent-difference scattering signal at the S_1_ minimum, at the QC minimum, and at the S_1_/S_0_ MECI, all relative to the NBD minimum. Since *I*(*t*,*q* → 0) = *N*_e_^2^, all signals start at zero. The individual geometries show similar patterns, with the two primary features being a peak at *q* = 1.5 Å^−1^ and a trough at *q* = 2.5 Å^−1^, which we take to be the signatures of the motion away from the NBD minimum. The percent difference becomes less pronounced at larger *q* as the signals approach their large momentum transfer limit, *I*(*t*, *q* → *∞*) = *N*_e_. In real experiments, this signal would be damped by the ‘excitation fraction’, the fraction of molecules that are excited by the pump pulse. For percentage difference, this acts as a global scaling factor of the signal strength.^28^

**Fig. 2:**
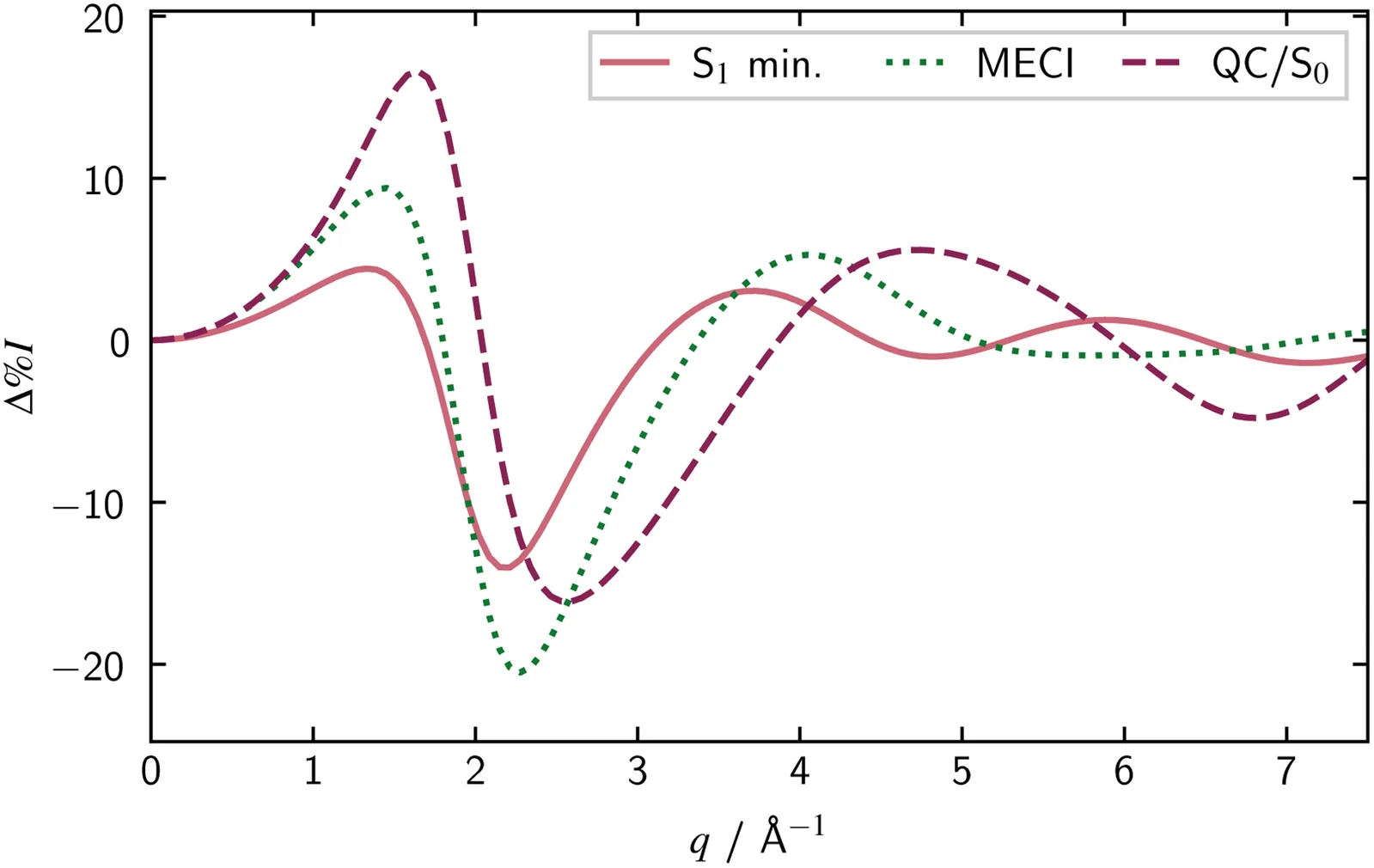
Percent-difference total X-ray scattering for the S_1_ local minimum (rose solid lines), the S_1_/S_0_ MECI (green dotted lines) and the QC ground state minimum (purple dashed). All three geometries are quite reminiscent of one another, reflecting that all involve distortion in a similar direction (see Fig. 1A). All patterns use the IAM for the scattering and geometries calculated using CASSCF(2,2).

We show the scattering patterns for each simulation (ensemble) in Fig. 3. At very early times, the patterns all have Δ*%I* ≈ 0, indicating their proximity to the reference structure, *i.e*. the NBD ground-state wavefunction. At *t* ≈ 30 fs, the wavepacket begins to decay to the ground state and to split into the final products. The CASSCF(2,2) and XMS-CASPT2(4,4) ensembles show an apparent downward tilt of the strong negative feature, clearly splitting into two features. XMS-CASPT2(2,2) shows this feature splitting *but* staying at higher momentum transfer values. This matches well with the results in ref. 49, with the XMS-CASPT2(2,2) ensemble showing significant differences at this point compared to the CASSCF(2,2) and XMS-CASPT2(4,4) models. Finally, the CASSCF(4,4) ensemble shows a near-oscillatory feature, reflecting the much longer excited state dynamics.

**Fig. 3:**
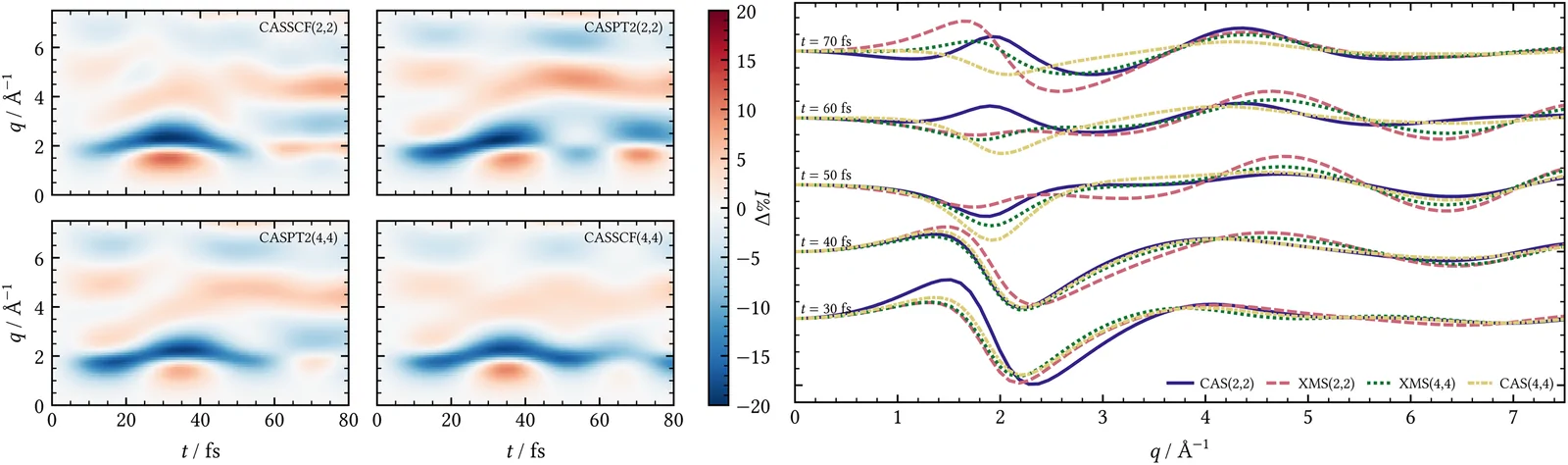
Percent-difference total X-ray scattering patterns for the four ensembles (simulations), calculated using eqn (31) and IAM. Left panel, heat-maps clockwise from top left: CASSCF(2,2), XMS-CASPT2(2,2), CASSCF(4,4), XMS-CASPT2(4,4). Right panel: Lineouts in time of the percent-difference signals, offset along the *y*-axis to separate the signals. All patterns share the same strong feature at around *t* ≈ 40 fs, corresponding to passage through the conical intersection, but differ at longer times due to different product quantum yields.

Towards the end of the simulations, the CASSCF(2,2) shows both lower signal strength and a weakly negative feature at *q* = 0.5 Å^−1^, an indication that the system returned to where it started in NBD. In contrast, XMS-CASPT2(2,2) shows a stronger overall signal and a positive feature at low momentum transfer, indicating the formation of QC, as borne out by the static patterns of Fig. 2. Pleasingly, the XMS-CASPT2(4,4) shows a signal somewhere in between, perhaps slightly favouring the more similar dynamics of the CASSCF(2,2).

We can quantify these features by analysis of the negative feature found at around *q* = 2 Å^−1^ and the smaller positive feature that appears at *q* = 1.5 Å^−1^, as shown in the lineouts of Fig. 4. Immediately, we notice the strong motion in the *q* ≈ 2 Å^−1^ region at early times, indicating distinct coherent motions away from the Franck–Condon (FC) region. This is mirrored in the *q* ≈ 1.5 Å^−1^ region (where positive indicates departure from the FC region).

**Fig. 4:**
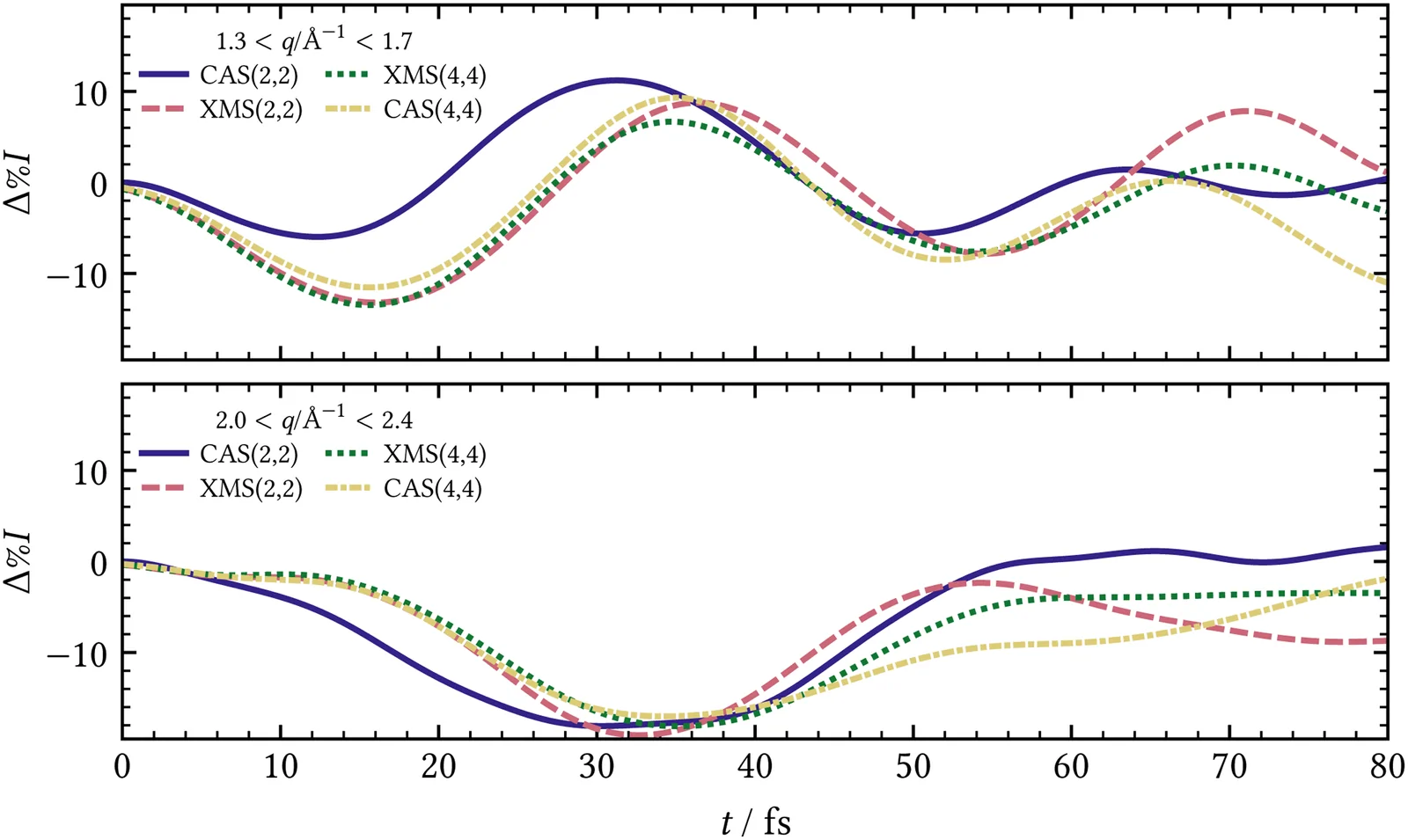
Lineouts integrated over small *q*-regions of the percent-difference scattering patterns in Fig. 3. Upper panel: for the lower-*q* feature with 1.3 < *q* < 1.7 Å^−1^. Lower panel: the feature with 2.0 < *q* < 2.4 Å^−1^, where negative features indicate motion from NBD towards QC (and correspondingly, the S_1_ minimum and the MECI).

At longer times, we can identify some of the features in the scattering with aspects of the simulations. For instance, the CASSCF(2,2) scattering returns to Δ%*I* = 0 in the two lineouts, indicating a return to the NBD minimum. The CASPT2(2,2), on the other hand, shows negative features in the *q* ≈ 2 Å^−1^ lineout. This is an indication of motion away from NBD, and further, according to Fig. 2, the formation of QC. The CASPT2(4,4) ensemble splits the two, as we know it should from inspection of the simulations. It is worth noting that specific structural changes give rise to intensity variations in the scattering across extended regions of the reciprocal space, and can be related directly to the distribution of molecular geometries.^114^ On the other hand, it is not trivial to distinguish the overlapping contributions from distinct channels and geometries in the scattering. Consequently, the amplitude of a given structural or electronic contribution is not always apparent from visual inspection, and quantitative analysis typically requires comparison with simulated basis signals or explicit fitting procedures.^115,116^

#### 4.1.1 Reaction channels

To confirm how the individual features arise, we plot the scattering patterns as separated into the aforementioned reaction channels in Fig. 5. We do not include the CASSCF(4,4) ensemble in this figure, as that simulation does not neatly conform to the same reaction channels as the other three simulations. The first column shows the NBD-ending trajectories for each of the different electronic structure methods, showing that the overall structure of these patterns is exceptionally similar, with the characteristic early time feature at ≈40 fs, followed by a shift of the negative feature to a lower *q*-value.

**Fig. 5:**
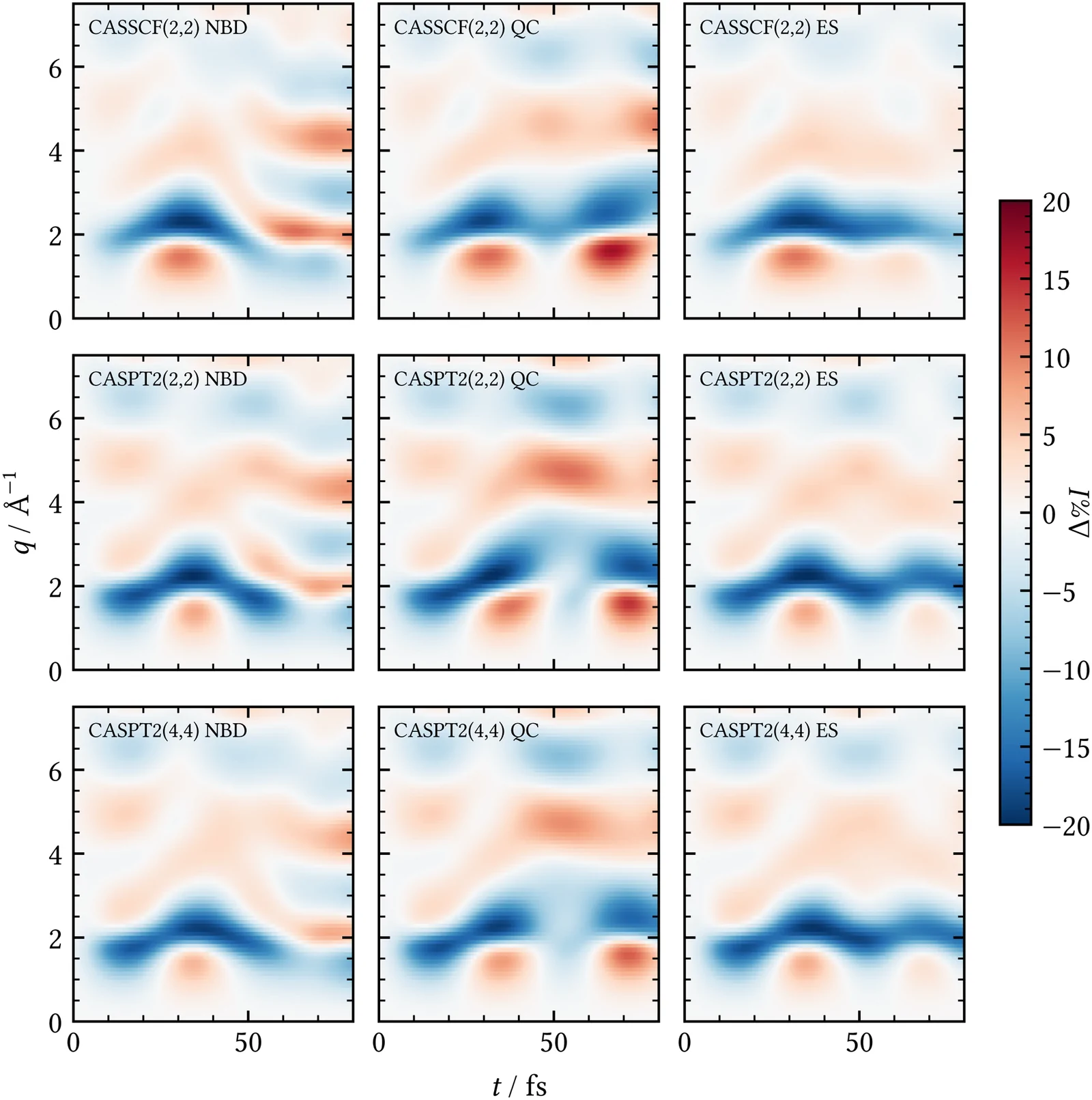
Percent-difference X-ray total scattering separated according to electronic structure method, calculated using eqn (31) and IAM (rows: CASSCF(2,2) top, XMS-CASPT2(2,2) middle, and XMS-CASPT2(4,4) bottom) and reaction channel (columns: NBD-ending left, QC-ending middle, and ES right). The differences between the reaction channels are clear throughout for the different dynamics ensembles, but the differences between electronic structure models within each reaction channel are more subtle.

The QC-yielding trajectories (middle column in Fig. 5) are notably different from the NBD-yielding, with the blue feature remaining at larger values of *q* even towards longer times. Finally, the ES trajectories show an oscillatory signal that indicates excited state dynamics, as seen earlier in the CASSCF(4,4) dynamics of Fig. 3.

We note that each of the three reaction channels is relatively similar across the three electronic structure methods considered here—*e.g*. the scattering patterns for the NBD trajectories are quite similar for all three electronic structure methods. This is somewhat less true for the QC trajectories, with a visibly different shape that could be read to reflect the subtly different mechanisms for each of the electronic structure methods.

### 4.2 Electron scattering

From the derivation of Section 2.2.1, it is clear that within the IAM approximation, the X-ray and electron scattering are closely related. In fact, essentially the same structural information is contained in both signals. This is borne out by the overall similarity of Fig. 3 and 6, which show the X-ray and electron scattering, respectively. The differences between the two are mostly in the intensity of the various features, and not in their overall shapes or locations in momentum transfer. As such, the results of the reaction-channel resolved electron scattering are very similar to the X-ray scattering, and we do not discuss them further here. This is not to say that there is no difference between the two experiments; this, however, comes mainly in the context of the ‘electronic effects’ discussed next.

**Fig. 6:**
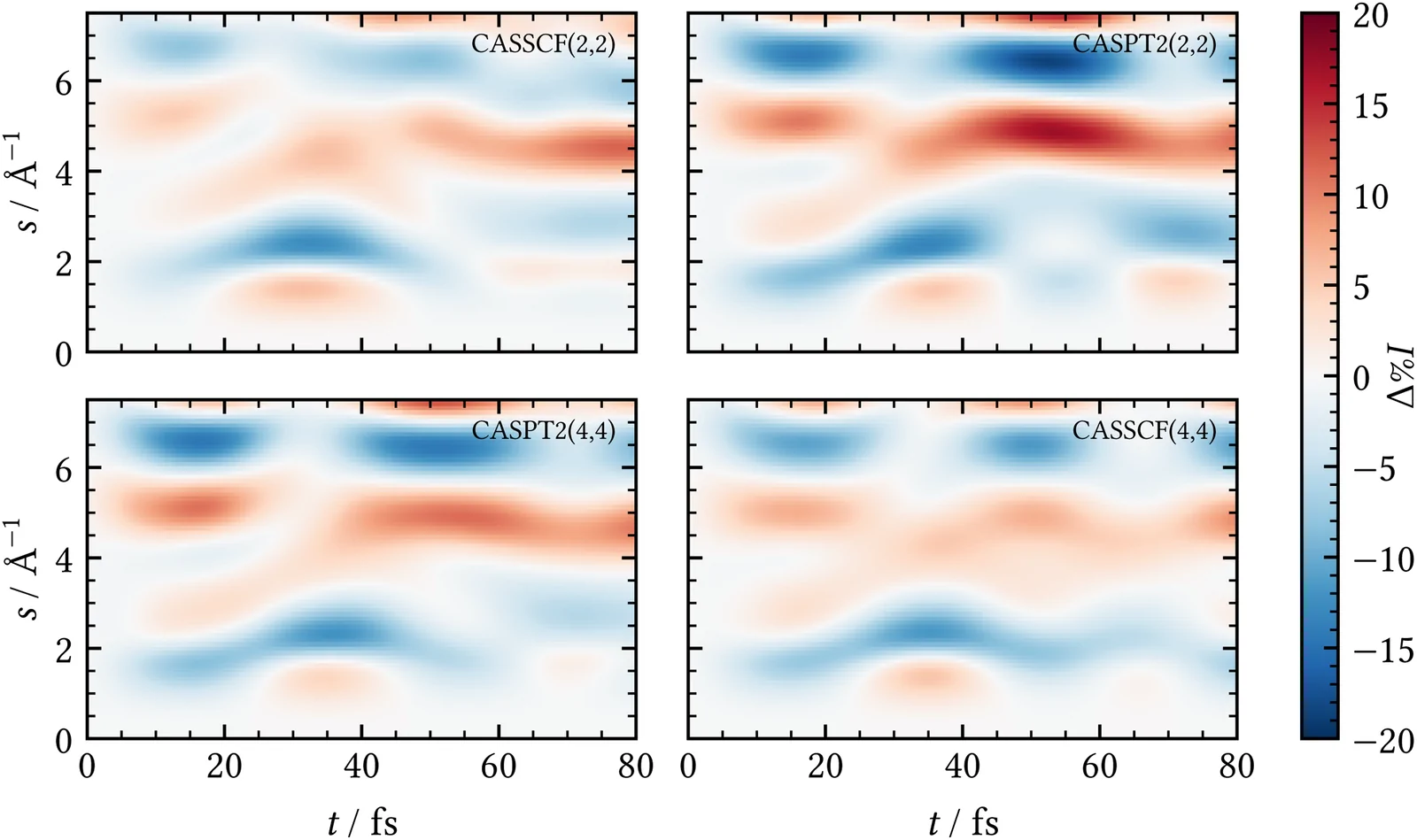
Electron scattering percent-difference signals for each of the four ensembles (simulations), calculated using eqn (31) and IAM and shown as heat-maps: CASSCF(2,2) (top left), XMS-CASPT2(2,2) (top right), XMS-CASPT2(4,4) (bottom left), and CASSCF(4,4) (bottom right). Analogue of Fig. 3.

### 4.3 Electronic effects in scattering

As discussed in Section 4.1, the X-ray total scattering cross-section is the Fourier transform of the two-electron pair-distribution function. Despite this, much of the signal is related to the nuclear positions, as the electron–electron distances are generally closely related to the interatomic distances due to tightly bound core electrons. The situation is similar in electron scattering, with two additional terms; an electron-nuclear cross term and a purely nuclear term, both containing explicit nuclear information.

To assess what is lost in the IAM approximation, we compare it with *ab initio* scattering calculations that use the electronic wavefunction explicitly (see further details in Section 3). We use these calculations to ask how large the electronic contributions to the scattering are in this case. The deviations between IAM and *ab initio* scattering relate to the absence of a state-specific electronic character in IAM, as well as the absence of chemical bonding, correlation, and charge transfer effects.

Fig. 7 compares three X-ray scattering signals for the same CASSCF(2,2) trajectory ensemble. The active-state *ab initio* signal calculates the scattering using the electronic wavefunction of the active state for each trajectory and thus represents our highest-level prediction. The ground-electronic-state *ab initio* signal in the next panel uses the exact same nuclear geometries, but calculates the scattering using the ground electronic state in each instance. Chemical bonding and correlation effects are thus quite similar in these two calculations, and the differences between them isolate the state-specific effect of the electronic character. Because these differences are very small, on the right of the figure we include two panels that show the double-difference scattering, defined as

32 ΔΔ%*I*(*t*,*q*) = Δ%*I*(*t*,*q*) − Δ%*I*^ref^(*t*,*q*),

*i.e.* the difference between a percent-difference pattern and our reference, the full *ab initio* active-state pattern.

**Fig. 7:**
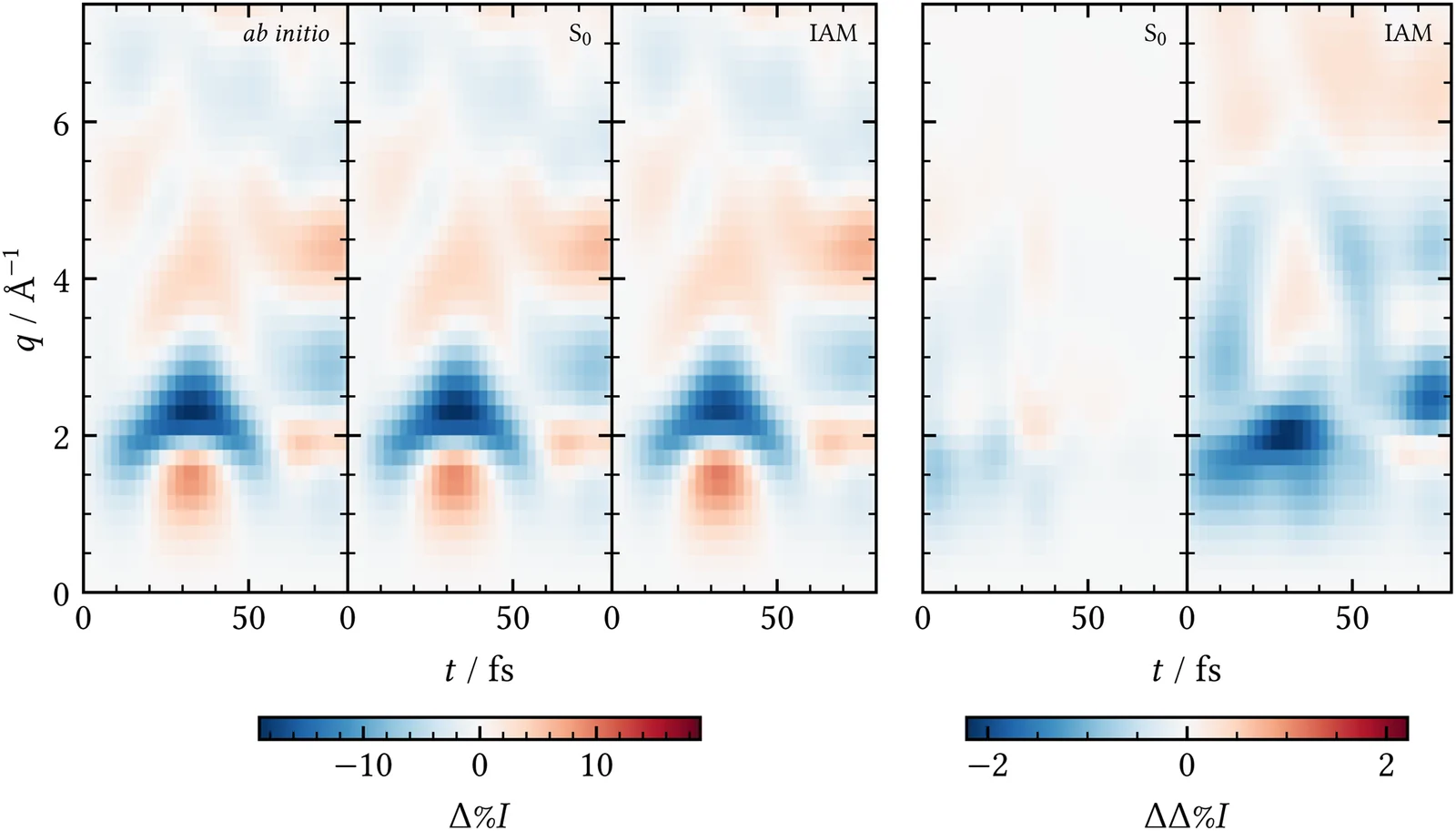
*Ab initio* and IAM total X-ray scattering for the CASSCF(2,2) ensemble (simulation). Left three panels: percent-difference signals calculated using (left to right), active-state *ab initio* scattering, ground-electronic-state *ab initio* scattering, and IAM. Right two panels: double-difference signals, eqn (32), relative to the active-state *ab initio* scattering; first panel is the double-difference with ground-electronic-state *ab initio*, second with IAM. The electronic state-specific difference in the first panel is small, while the IAM deviation in the second panel is larger but remains around approximately ±2%.

The active-state minus ground-electronic-state double difference is small throughout, well below 1%. Thus, for this NBD/CASSCF(2,2) case, state-specific effects make only a minor contribution to total X-ray scattering. The difference is largest at early times, when the excited-state population is at its largest, and fades as the ensemble returns to the electronic ground state, where the double difference equals zero by definition.

The IAM double difference is larger, reaching slightly above 2%, or more than 10% of the total percent-difference variation. The largest deviations occur around 40 fs, when the trajectories approach the conical-intersection region. This is consistent with the IAM being least reliable for strongly distorted geometries where bonding and electronic structure are changing rapidly. Even so, the corrections remain secondary to the dominant structural signal.

Fig. 8 shows the analogous comparison for electron scattering. We note that, just like the IAM, the *ab initio* signals have the same features as the X-ray scattering results, with a scaling of the intensities. In contrast to X-ray scattering, the IAM deviations are much more prominent, especially at low *s* where both *ab initio* signals show large differences to the IAM. Such low-*s* features are often interpreted as electronic excitation,^37^ but the present decomposition shows that this interpretation requires some care, as a similar signal is also observed in the pure ground state *ab initio* signal (labelled S_0_). Thus, in this case, the low-*s* post-IAM signal cannot be assigned to excited-state population alone. A substantial contribution comes from changes in bonding and charge density as the molecule distorts toward the CI region, an effect absent from the IAM.

**Fig. 8:**
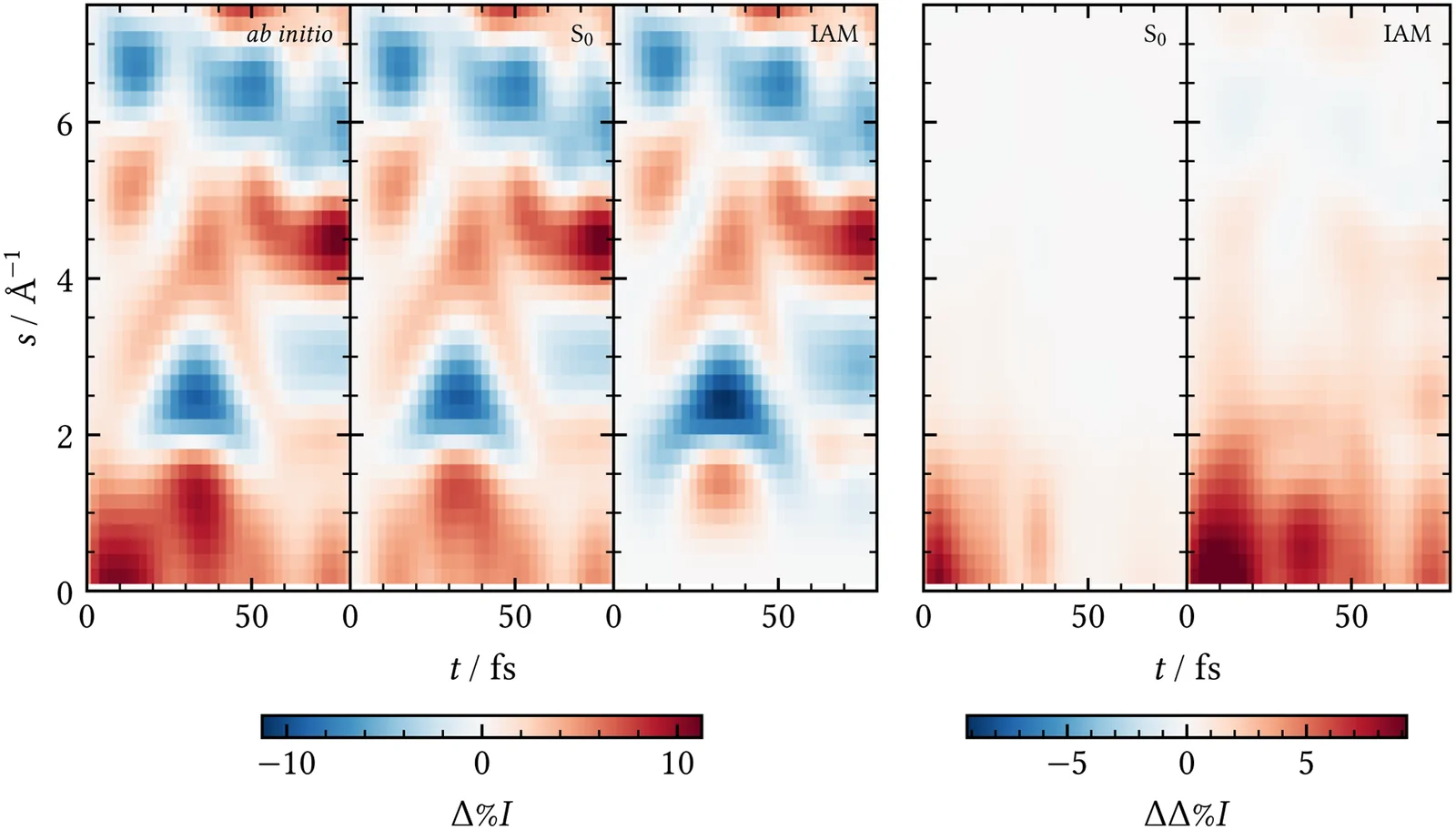
*Ab initio* and IAM electron-scattering signals for the CASSCF(2,2) ensemble (simulation). Left three panels: percent-difference signals calculated using (left to right) active-state *ab initio*, ground-electronic-state *ab initio*, and IAM. Right two panels: double-difference signals, eqn (32), relative to the active-state *ab initio* result; first panel is the double-difference with the ground-electronic-state *ab initio*, and second with IAM. The first *s* point is omitted because of numerical noise in the denominator of eqn (31).

Finally, we note that the apparent relative magnitude of the corrections depends on the percent-difference representation. For X-ray scattering, the reference signal in the denominator is large, approaching *N*_e_^2^ at low *q*, so electronic and post-IAM corrections appear diminished in the percent-difference. For electron scattering, the opposite can occur: the *s*^−4^ prefactor is removed by the division in eqn (31), and the remaining reference signal becomes small at low *s* (for neutral systems), so modest absolute differences can appear strongly amplified in the percent-difference map. This difference in normalisation partly explains why the post-IAM effects appear much more dramatic for electron scattering than for X-ray scattering in Fig. 7 and 8, while from eqn (10) and (15)–(18) it can be seen that the absolute differences are relatively similar in magnitude. Thus, while these figures identify where the IAM and *ab initio* predictions differ for this NBD/CASSCF(2,2) case, a more general assessment of experimental measurability would require absolute signals, realistic detector ranges, experimental normalisation procedures, and signal-to-noise modelling. Further discussion of X-ray *versus* electron scattering can be found in ref. 84, 98 and 99.

## 5 Photoelectron spectroscopy

For photoelectron spectroscopy, to be consistent with experiments^39,40,118^ we shall show the difference signal

33 Δ*σ*_PES_(*t*,*E*_BE_) = *σ*_PES_(*t*,*E*_BE_) − *σ*_PES_(*t* ≪ 0,*E*_BE_),

where *σ*_PES_(*t*, *E*_BE_) is the photoelectron spectrum for a given time *t* and binding energy *E*_BE_, with *σ*_PES_(*t*, *E*_BE_) calculated using eqn (25). The expression in eqn (33) removes the signal from the non-excited portion of the initial ground state wavefunction, which is static in the limit of a one-photon excitation process. Therefore, the spectra will show two sets of features with opposite signs: the excited state signal, which gives a positive signal, and the ground state bleach signal, which gives a negative. As in scattering experiments, the size of this signal is modulated by the excitation fraction, which we do not account for here. Later, we shall show the signal split into both individual reaction (Section 5.3.1) and ionisation channels (SI), using the plain *σ*_PES_(*t*, *E*_BE_) from eqn (25) for simplicity.

### 5.1 Nature of the ionic potential energy surfaces

In Fig. 9, we show the potential energy cuts for the ionic states calculated using the Δ-method described in Section 3, using the high-level IP-LR-CC3 as a reference. We first note the two states in CASSCF(1,2) (left panel, solid indigo lines), with a relatively flat state at ≈7 eV and a very steep state which lies between 14 and 9 eV across the plot. These states represent the two states made from the |2u00〉 and |20u0〉 configurations, with the former making up D_0_ on the right of the pathway and D_1_ on the left.^78,117^ These two states are Koopmans’ allowed from the S_1_ state (of |2ud0〉 character).

**Fig. 9:**
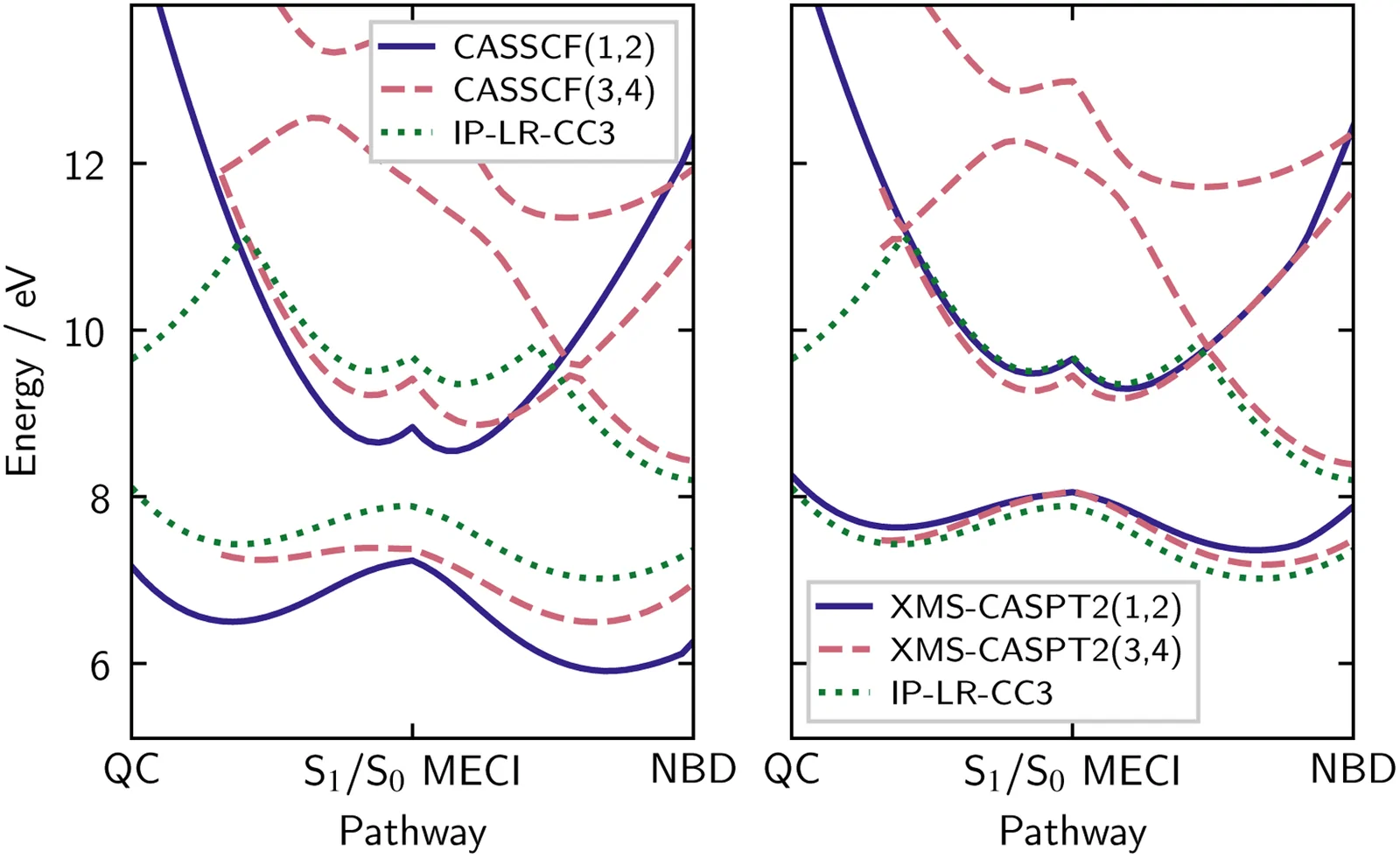
Ionic potential energy surfaces on the QC → S_1_/S_0_ MECI → NBD LIIC (see Fig. 1D and ref. 81). For each, the energy zero has been set at the QC neutral minimum. Left: CASSCF(1,2) (solid indigo lines) shows two states, the lower relatively flat at 7 eV and the higher changing much more rapidly, which corresponds to D_2_ in Table 1. CASSCF(3,4) (rose dashed) shows two more states, corresponding to the D_1_ and D_2_ in Table 1. IP-LR-CC3 (green dotted, both panels) shows a similar lower state but requires more roots to find the additional states. Right: The XMS-CASPT2 [(1,2) indigo solid, (3,4) rose dashed] and IP-LR-CC3 calculations all agree strongly when they find the same states, but there are additional channels in XMS-CASPT2(3,4) not seen in the other methods. As in ref. 81, we do not show the results for the (4,4) active space near to QC, where it is unstable.

To understand the other doublet states, we need to move to a method with more flexibility—XMS-CASPT2(3,4). The potentials for XMS-CASPT2(3,4) (dashed rose lines, right panel of Fig. 9) clearly show the two states of |2u00〉 and |20u0〉 character and add a low lying D_1_ state in NBD, with rising energy. We include the binding energies, cross-sections, and configurations for the four states in Table 1, as well as reference IP values for both isomers. Here, we can see that D_1_, with |u200〉 character, cannot be described in the CASSCF(1,2) calculations. Fortunately, this state is Koopmans’ forbidden from the S_1_ state, and so should not significantly affect the predicted signal from the excited state dynamics.

**Table 1:** Ionic states, calculated at the NBD geometry using ΔXMS-CASPT2(3,4)/p-cc-(p)VDZ, using XMS-CASPT2(4,4) for the neutral states. Ionisation potentials (Δ*E*) and Dyson norm cross-sections are shown for ionisation from S_0_ and S_1_. The wavefunction character is shown in occupation number representation (|*η*_1_*η*_2_*η*_3_*η*_4_〉, with orbitals shown in ref. 81), with weights over 20% shown. States whose leading character can be described in the (2,2)/(1,2) active space are distinguished in the final column. For reference, the IP-LR-CC3 S_0_ → D_0_ ionisation potentials at the ground-state minima are *E*_IP_(NBD) = 8.51 eV and *E*_IP_(QC) = 8.11 eV, in good agreement with experiment^117^

Δ*E*/eV			Character	Cross-section		
State	S_0_	S_1_		S_0_	S_1_	In (1,2)?
D_0_	8.42	3.42	|2u00〉	0.93	0.47	Yes
D_1_	9.33	4.32	|u200〉	0.94	0.03	No
D_2_	12.61	7.61	0.66|20u0〉 + 0.21|02u0〉	0.03	0.30	Yes
D_3_	13.31	8.30	0.47|200u〉 + 0.37|uud0〉	0.05	0.32	No

We finally note that, due to the small active spaces used, these are not complete descriptions of all possible ionic states. While this implies that we exclude certain ionisation channels, the important (high cross-section) channels are included and the less important channels all occur at higher energy, well above the ground state ionisation potential of ≈8.5 eV. As transitions larger than the ground state ionisation potential (IP) are more challenging to observe in experiments due to congestion, we can be confident that we have the qualitatively correct spectrum.

### 5.2 Time and energy resolution

Before we dive into the actual appearance of the signals and how they relate to the dynamics, we first examine the effect of the experimental resolution. Thus, we aim to find the probe pulse duration that maximises the contrast in the time-resolved photoelectron spectra. We test four different pulses, detailed in Table 2. Here (and later), we look at two aspects; the overall two-dimensional time-energy heat-map, and a ‘lineout’—the signal integrated between 3.5 and 4 eV.

**Table 2:** Probe pulse energy bandwidth Δ*E*_pr_ and overall experimental time-resolutions Δ*t* (eqn (27)) for a given probe pulse duration Δ*t*_pr_, with an excitation pump pulse duration of Δ*t*_pu_ = 6.71 fs. All quantities are full-widths-at-half-maximum (FWHMs) and assume Gaussian pulse envelopes

Δ*t*_pr_/fs	Δ*E*_pr_/eV	Δ*t*/fs
1	1.82	6.78
6.71	0.27	9.49
20	0.09	21.10
40	0.05	40.56

Immediately, it is clear that the shortest probe pulse duration (Δ*t*_pr_ = 1 fs, upper left panel in Fig. 10) is problematic for two reasons: the energy spread due to the pulse (Δ*E*_pr_ = 1.82 eV) is large, and the overall ≈7 fs time-resolution limit due to the pump remains. At the other end of the scale, the longer duration 20 and 40 fs pulses (Fig. 10, bottom left and right panels respectively) conceal detail as much of the interesting dynamics occurs on shorter time-scales than their time-resolution. The intrinsic ≈0.3 eV bandwidth of the pump pulse further negates some of the benefit of the more narrow bandwidth probes.

**Fig. 10:**
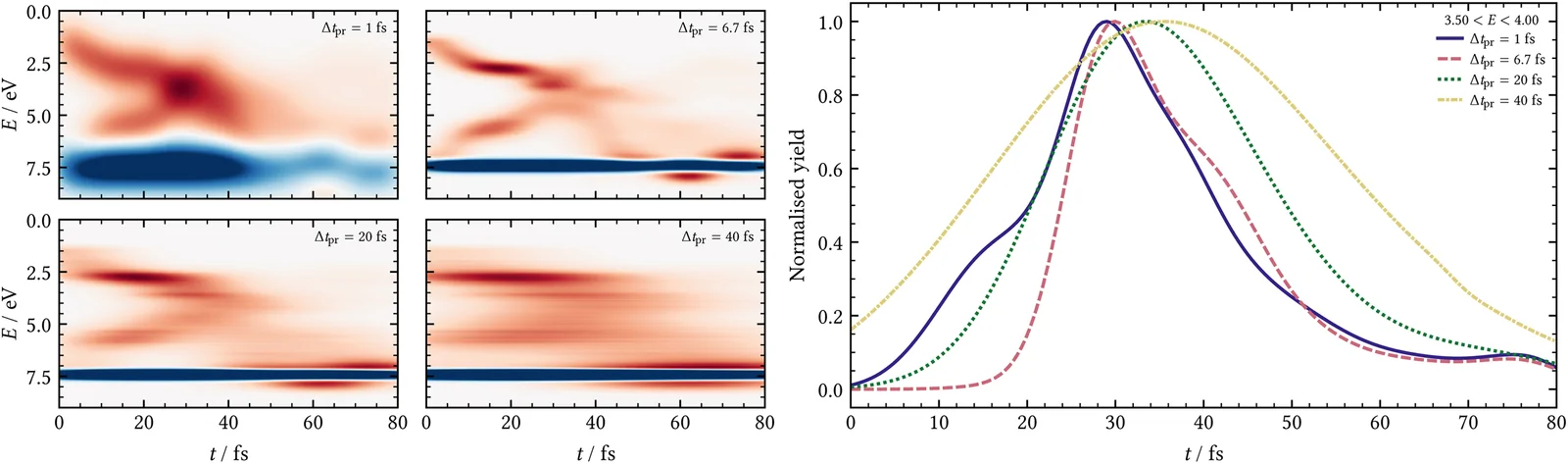
Time-resolved photoelectron spectra as a function of time and binding energy, calculated using eqn (33), with the different probe pulse durations listed in Table 2. All calculations are for CASSCF(2,2) dynamics and use the Dyson norm approximation. Left panels: The shortest probe pulse (upper left, Δ*t* = 6.78 fs, Δ*t*_pr_ = 1 fs) and longer probe pulses (bottom left panel, Δ*t* = 21.10 fs, Δ*t*_pr_ = 20 fs and bottom right panel, Δ*t* = 40.56 fs, Δ*t*_pr_ = 40 fs) show little structure. The upper right panel shows the Δ*t* = 9.49 fs, Δ*t*_pr_ = 6.71 fs probe pulse, which matches the resolution of the pump pulse, and shows detail in both energy and time. The individual spectra have been normalised to aid comparison. Right panel: a lineout of the signal, integrated between 3.5 and 4 eV, for the various pulses. Interestingly, the fastest probe pulse does not lead to the best time-resolution, as the poorer energy resolution masks some features. The best overall resolution is obtained for the Δ*t*_pr_ = 6.71 fs pulse.

The favourable middle ground is found when the pump and the probe pulses have the same duration, Δ*t*_pr_ = Δ*t*_pu_ (top right panel, Fig. 10). This spectrum exhibits the greatest contrast, with sufficient time and energy resolution to pick out relevant features.¶¶Δ*t*_pr_ = Δ*t*_pu_ always gives the highest combined resolution, as the overall time-bandwidth product is 
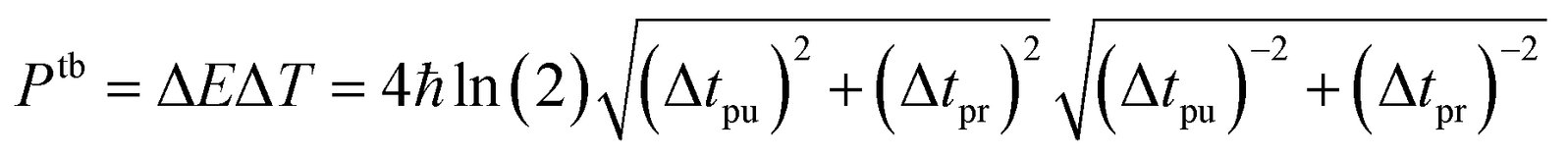
, using the equality in eqn (26) and (27). This function is minimal (*P*^tb^ = 8*ħ*ln(2)) at Δ*t*_pr_ = Δ*t*_pu_. Although we only show results for the CASSCF(2,2) simulations, the other ensembles yield very similar results in this analysis, and we use the Δ*t*_pr_ ≈ 6.71 fs probe pulse for all time-resolved photoelectron spectra (TRPES) data shown henceforth.

We note that for dynamics similar to those investigated here, it appears that pulses in the vicinity of Δ*E* ≈ 0.3 eV, Δ*t* ≈ 10 fs appear to constitute a reasonable compromise. The energy resolution is sufficient to detect significant changes as the molecule undergoes bond breaking, while the temporal resolution is sufficient to capture all but hydrogen-atom motions. Significantly shorter or longer pulses reduce either the time or energy resolution, obscuring the dynamics either way. Experimentally, there is rapid progress in this area at the moment and sub-10-fs pulses appear increasingly achievable.^41,119^

### 5.3 Electronic structure methods

The simulated time-resolved photoelectron spectra (TRPES) are shown in Fig. 11, according to the electronic structure method used to propagate the trajectories. We first note the similarity between the CASSCF(2,2) and the two XMS-CASPT2 signals. There are two principal features, one decreasing and one increasing in binding energy as we move beyond *t* = 0. At the CI (≈40 fs), these two features meet, and then the majority of the signal proceeds to around *E*_IP_ = 8 eV, indicative of the formation of ground-state NBD and QC (see Table 1). We also note that the ground-state bleach, the strong negative feature caused by excitation out of the ground state, is lower in binding energy in the CASSCF(2,2) calculations. This merely reflects that the energy of the D_0_ is too low, as seen in Fig. 9, but does not indicate different chemistry.

**Fig. 11:**
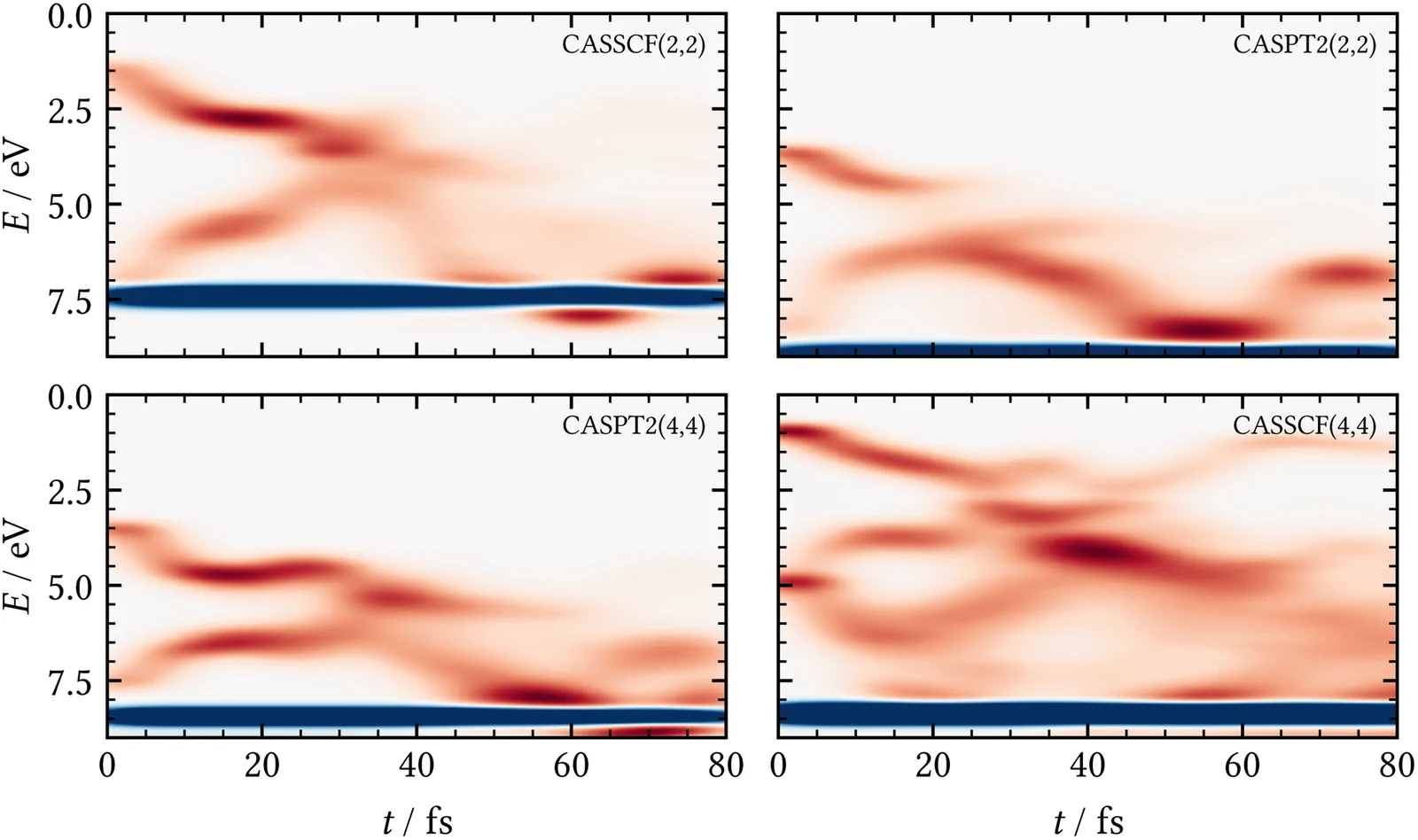
Time-resolved photoelectron spectra for each of the four ensembles (simulations), calculated using eqn (33) and shown as a function of time and binding energy. The two (2,2) active space methods (upper panels) show similar structure, albeit with a notable shift in binding energy. The XMS-CASPT2(4,4) (lower left) shows a few additional features while still resembling the (2,2) methods, with CASSCF(4,4) (lower right) adding even more features. The figures have been normalised to aid comparison. All spectra show a similar double feature at early times, with a low-binding energy and a high-binding energy feature moving together and crossing, signalling passage through the conical intersection (CI).

From these plots, the overall time-scale of the dynamics is apparent. For example, the CASPT2(2,2) dynamics gives a crossing of the two early features at around 25 fs, earlier than in CASSCF(2,2) and CASPT2(4,4). We can quantify this with the processed ‘lineouts’ shown in Fig. 12, which are calculated using34
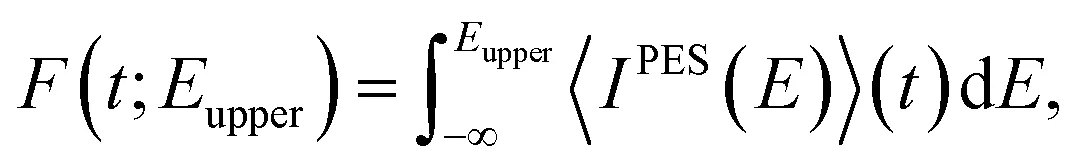
which is then renormalised such that the maximum becomes one. These lineouts quantify the amount of signal below a given binding energy *E*_upper_, which we set at 4.5 eV for the CASSCF and 6.5 eV for the CASPT2. We note that small changes (±0.25 eV) of *E*_upper_, as depicted by the shaded regions in Fig. 12, do not significantly change the results.

**Fig. 12:**
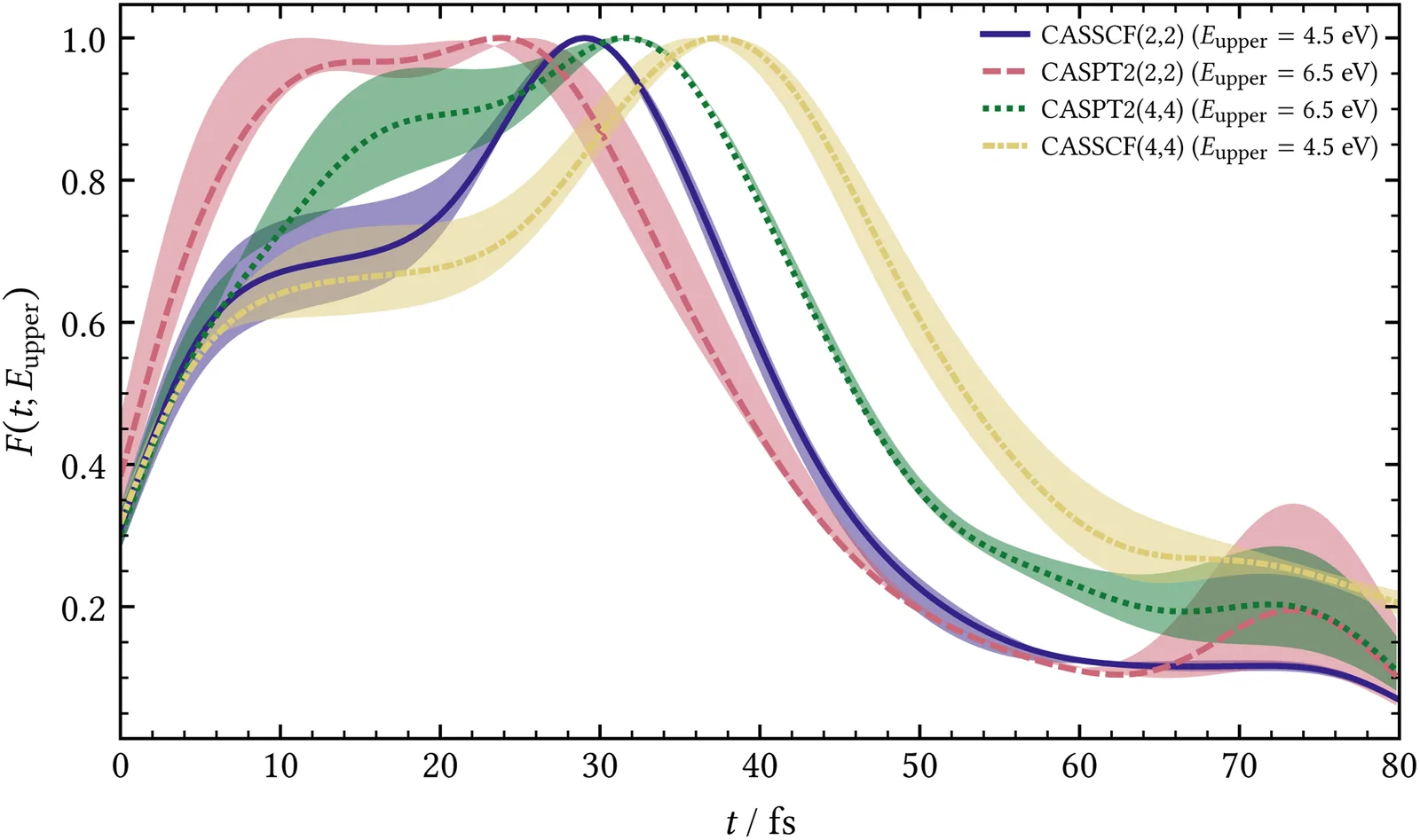
Processed lineouts in time calculated using *F*(*t*; *E*_upper_) in eqn (34), which integrates all values below *E*_upper_ (listed for each ensemble in the legend). These lineouts are shown for all four ensembles (simulations) in Fig. 11. For each trace, the shaded region indicates the values found by *E*_upper_ ± 0.25 eV. The CASSCF(2,2) and CASPT2 ensembles give decays in good agreement with Fig. 1C, while CASSCF(4,4) shows a longer decay. The small increases at long times in the CASPT2 simulations are due to hot ground state dynamics.

Looking closely at Fig. 12, we can see that the time at which *F*(*t*; *E*_upper_) begins to drop off correlates strongly with the beginning of the population decay (see Fig. 1C), at least for the first three methods. Indeed, we recover the correct ordering of decay lifetime, as well as approximately correct lifetimes.

The CASSCF(4,4) dynamics, on the other hand, shows a relatively strong signal at lower binding energies and at later times, as can be seen clearly in Fig. 11. This provides the clearest spectroscopic signature of the longer-lived excited-state population in the CASSCF(4,4) ensemble, and the additional peaks indicate the presence of extra channels, in this case due to the mixture of S_1_ and S_2_ dynamics.

The final product in the dynamics can be recognised in Fig. 13, which shows lineouts of the energy at *t* = 70 fs. Here, for the first three models the dynamics is almost entirely in the ground state, whereas excited state dynamics is still present in CASSCF(4,4). This is immediately evident from Fig. 13—CASSCF(4,4) has a lot of features at low binding energy, whereas the other three have much weaker signals.

**Fig. 13:**
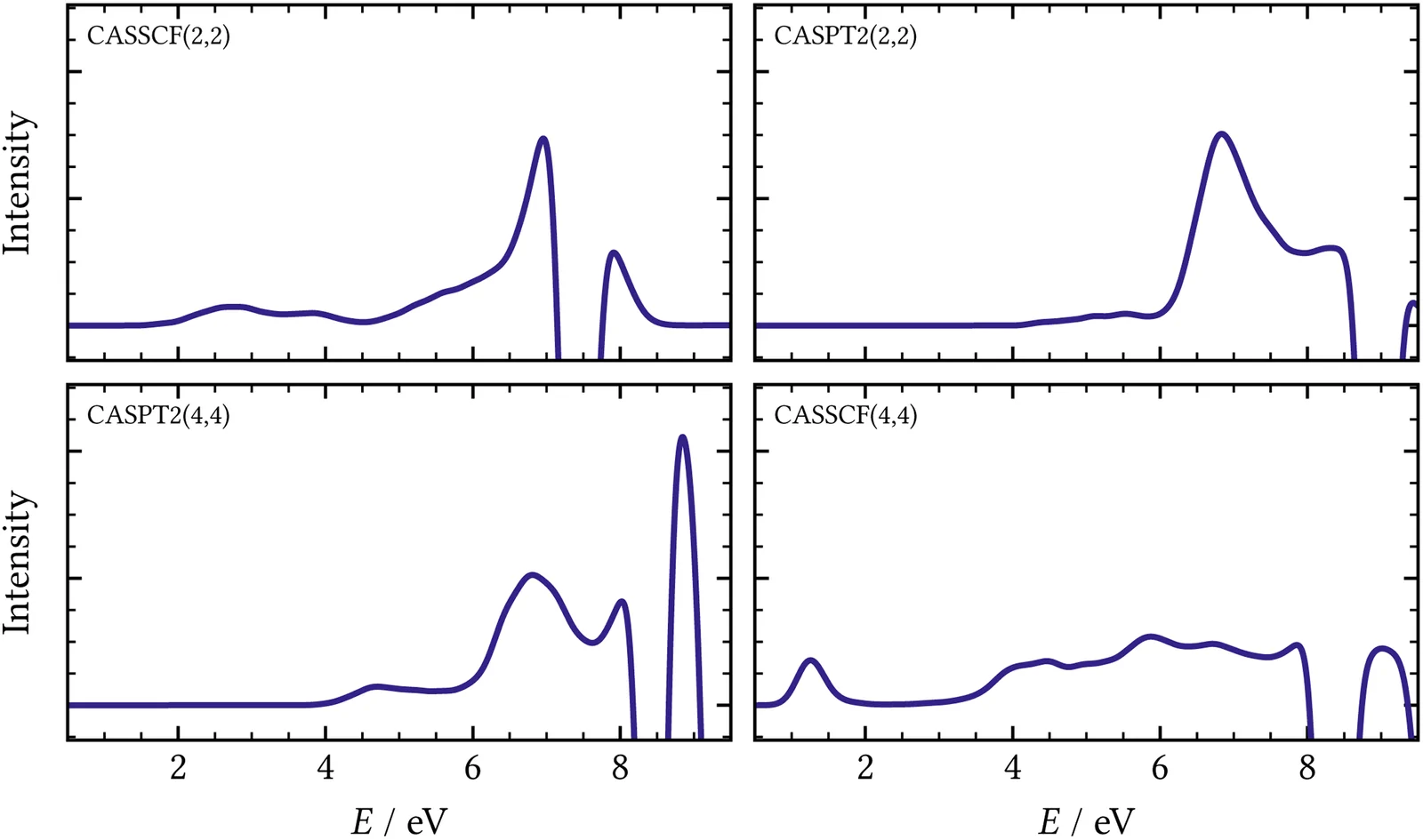
Lineouts of the photoelectron spectra in Fig. 11 at *t* = 70 fs for each ensemble (simulation), shown as a function of binding energy. In each plot, the ground-state ionisation potential (IP) of NBD can be inferred by the presence of the ground state bleach (the strong negative signal), and QC is known to be situated approximately 0.5 eV below that.^117^ The CASSCF(2,2) (top left) shows a broad signal around the NBD IP (indicating hot NBD formation) and a small shoulder in the QC region, while XMS-CASPT2(2,2) (top right) shows a strong QC signal and a signal around the ground-state bleach. The XMS-CASPT2(4,4) shows both of these signals strongly. The CASSCF(4,4) still shows strong signal much lower in energy than the ground-state bleach, indicating long-lived excited state dynamics.

Further, some information about the branching ratios can be inferred; as the ground state products are hot, they show significantly broadened peaks, and thus NBD formation is identified as a feature surrounding the ground state bleach. QC, however, has an IP approximately 0.5 eV lower in energy than NBD^117^ (see Table S1, SI), and its formation can be seen with a strong peak in that region, most obviously in the CASPT2(2,2) dynamics—as expected from the large product yield of that particular method. However, calculation of the branching ratio would be more complicated in an experiment. Merely integrating these peaks gives a relative measure, but it is weighted by the photoionisation cross-section. Therefore, extracting branching ratios requires theoretical predictions of the ionisation cross-sections, a non-trivial calculation.^102,103,105^ In the data presented here, we use the Dyson norm approach, which does not make quantitative predictions.

#### 5.3.1 Reaction channels

Fig. 14 shows the photoelectron spectra split into individual reaction channels. As before, we do not include the CASSCF(4,4) simulation, as it does not conform to the same categories as the other simulations. Immediately, we see that the separation between methods (*i.e*. down the columns) is similar to the separation between pathways (*i.e*. along the rows). This contrasts strongly with the scattering data discussed earlier.

**Fig. 14:**
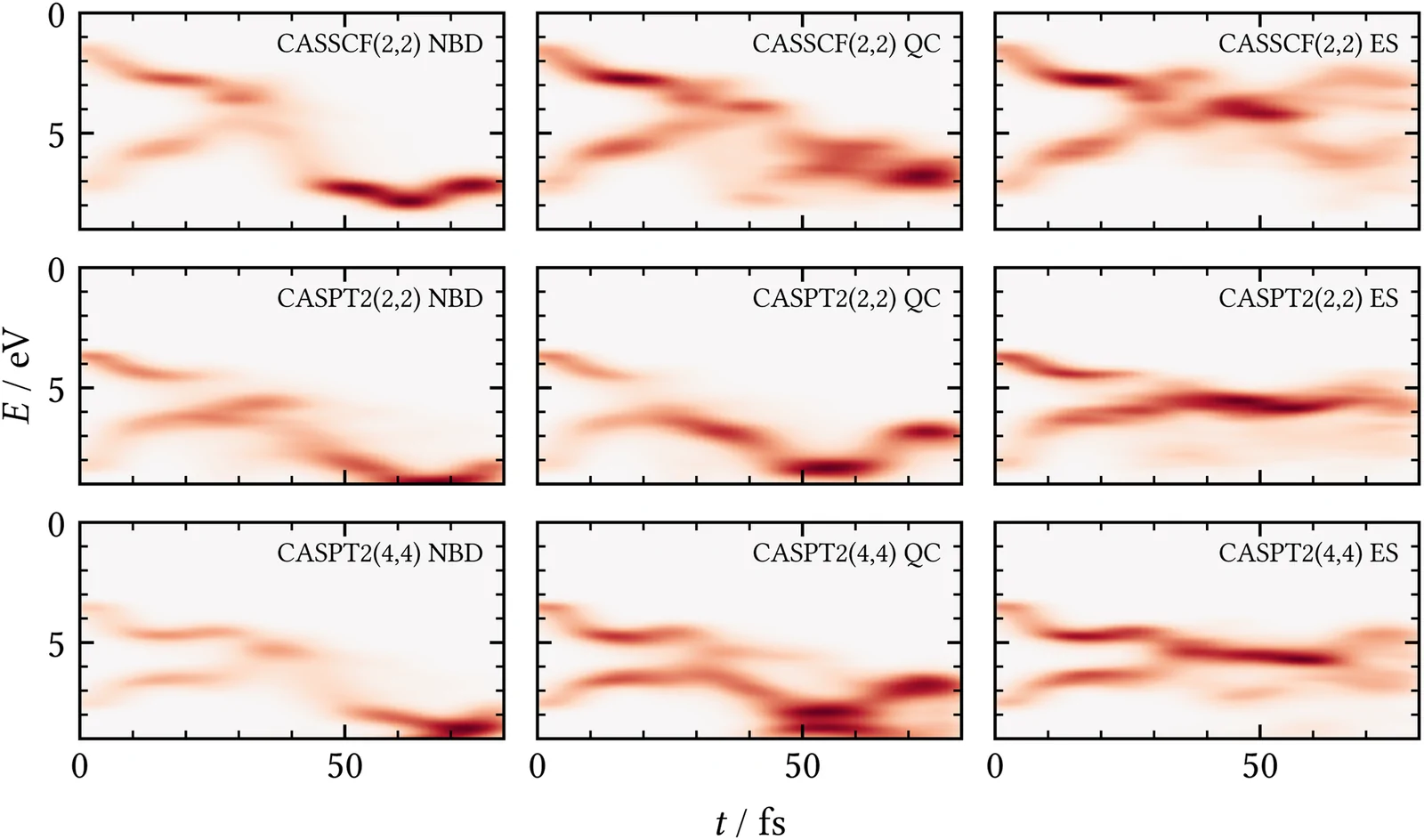
Time-resolved photoelectron spectra shown for the ensembles (simulations) split according to reaction channels. Top row: CASSCF(2,2). Middle row: XMS-CASPT2(2,2). Bottom row: XMS-CASPT2(4,4). Left column: NBD-forming trajectories. Middle column: QC-forming trajectories. Right column: ES trajectories. As opposed to Fig. 5, the differences between the different reaction channels are similar to the differences between the electronic structure methods. For clarity, the cross-sections have been re-normalised so that each panel’s maximum signal is identical to one.

The first column shows the predicted TRPES signals for the NBD-forming channel. This channel remains coherent for all ensembles, with a single path leading to strong oscillations in ground-state NBD (at ≈8 eV, ≈60 fs). The final oscillation is somewhat obscured in Fig. 11 by the ground-state bleach, which is also due to ground-state NBD.

The QC pathway (centre column, Fig. 14) shows a bit more structure. For the CASPT2(2,2), there is only one coherent channel, which forms ground state QC rapidly upon photoexcitation.^49^ This gives a single, relatively clean channel with little dispersion. For the other two models, we see some evidence of multiple features following passage through the CI (≈40 fs), matching the observed dynamics in previous work.^49^ We can also see that the ground-state binding energy is lower than the NBD-yielding trajectories, as discussed earlier.

Finally, the ES trajectories (right column, Fig. 14) show most of their signal at 6 eV or lower binding energies, at notably lower binding energies than the corresponding QC and NBD channels, as expected. Some trajectories exhibit larger binding energies, either from exploring a low-energy part of the potential, or by having a strong absorption to higher doublet states.

## 6 Conclusions

We have examined X-ray and electron scattering and photoelectron spectroscopy signals, calculated for four distinct excited norbornadiene dynamics. The scattering signal carries structural information, distinguishes between reaction channels, and could potentially measure quantum yields or branching ratios. However, the differences between the four different models for the dynamics are comparatively small, revealing a lack of sensitivity to subtle changes in the dynamics. The sensitivity to the time-scale of the reaction is also not that strong, with all four models predicting quite similar rates. However, these conclusions are somewhat system-dependent, and the sensitivity when the dynamics involves bond-breaking or dissociation is far greater.^28,36,120^

In addition, we have investigated the electronic effects in the scattering by comparing to the *ab initio* scattering patterns calculated for the CASSCF(2,2) model. This reveals distinct deviations in the scattering signal due to the absence of chemical bonding effects in the independent atom model. Notably, the specific excited-state contribution to the scattering is weak and mostly present at early times when excited state populations remain large. To determine the character of the excited state would thus require excellent data quality, or at least rather distinct scattering signatures, such as for Rydberg or charge-transfer states.^95,96^

Conversely, it is challenging to extract vibrational (structural) dynamics from photoelectron spectra. As we only have one variable (the binding energy) that changes significantly throughout the dynamics, the signals from different nuclear geometries overlap, making it hard to discriminate between them. TRPES may be able to provide some guidance into the ground-state product ratios, but only when the products have sufficiently distinct ionisation potentials and when signals rise above the ground-state bleach. We also note that measuring quantum yields requires knowledge of the photoionisation cross-sections, the accurate prediction of which is challenging.

This being said, TRPES is excellent for revealing electronic information. The lowering in binding energy as the wavepacket evolves is apparent from all plotted spectra, which makes it easy to parse the time-scale of the relaxation. One can even attempt to assign the passage through the CI *via* the crossing of the S_1_ → D_0_ and S_1_ → D_1_ features. Improving on the calculation of the photoelectron ionisation cross sections, for instance using the R-matrix method,^103,106^ would yield more quantitative predictions of the TRPES experimental signals. Such work could better account for the specific lineshapes associated with ionisation. Imaging, which would involve measuring (and calculating) photoelectron angular distributions, could plausibly be used to distinguish the individual ionisation channels and gain more information about the character of the electronic states involved in the dynamics, further validating or disproving the models considered here. We have also examined how the duration of the UV probe pulse influences the time and energy resolution of the recorded spectra, finding that pulses of a duration well matched to the dynamics (and the pump pulse) benefit the quality of the measurement.^121,122^ It would be interesting, in future work, to examine further observables in the same systematic fashion, for instance, X-ray absorption spectroscopy or nonlinear spectroscopies.^123,124^

In conclusion, information is lost when projecting the dynamics onto a single observable. Scattering reveals unmistakable details about the structural changes and the relative yield of the two products appears clearly, but electronic effects are more challenging to resolve and nontrivial to interpret correctly. On the other hand, the photoelectron spectroscopy captures the overall electronic decay very well. Our results thus strongly support the utility of combining spectroscopic and scattering measures – when combined, these stand a good chance of distinguishing competing theoretical models and suggest characteristics of the dynamics that can be determined based on experiments alone. Multimodal measurements, highly demanding of resources as they are, constitute a promising route for mapping and understanding excited state processes, and have the capacity to truly challenge and complement theoretical models.

## Author contributions

J. C. Cooper: conceptualisation, methodology, computations, software, formal analysis, investigation, writing, visualisation. A. Kirrander: conceptualisation, methodology, formal analysis, writing, supervision, funding acquisition.

## Conflicts of interest

There are no conflicts to declare.

## Supplementary Material

FD-OLF-D6FD00085A-s001

## Data Availability

Further information in support of this article is included in the supplementary information (SI). The source data for the figures is available from Figshare [https://doi.org/10.6084/m9.figshare.33029534]. Further data is available from the authors upon reasonable request. Supplementary information: decomposition of the photoelectron spectra into ionisation channels. See DOI: https://doi.org/10.1039/d6fd00085a.
