# Supplementary material for: The information content of ultrafast observables: spectroscopy and scattering in excited norbornadiene
Source: Faraday Discuss. 2026 Nov 11. Online ahead of print. doi: 10.1039/d6fd00085a (PMC13752845; doi:10.1039/d6fd00085a)
Supplement: Supplementary file 1 [file FD-OLF-D6FD00085A-s001.pdf]

Supplementary Information (SI) for Faraday Discussions.
This journal is © The Royal Society of Chemistry

Supplementary Information
The Information Content of Ultrafast Observables: Spectroscopy and Scattering in Excited Norbornadiene

J. C. Cooper and A. Kirrander

Physical & Theoretical Chemistry Laboratory, Department of Chemistry, University of Oxford, South Parks Rd., Oxford, UK, OX1 3QZ

1 Photoionisation Channels

In this section, we break down the spectra into separate ionisation channels, defined by an initial (neutral) and final (ionic) state (e.g. $S_1 \to D_0$ indicates the channel from the first excited neutral singlet state to the ground ionic doublet state). In principle, these different signals are all observed simultaneously on the detector.[1] In a theoretical calculation, we can separate these channels to interpret the makeup of the total spectrum. For clarity, we will only show strongly contributing channels and again drop the analysis of CASSCF(4,4).

In Fig. S1, we show this separation for the CASSCF(2,2) dynamics. The aforementioned low- and high-binding-energy features arise from the $S_1 \to D_0$ channel (top left panel in Fig. S1) and $S_1 \to D_1$ channel (top right panel in Fig. S1). Their meeting at $t = 40$ fs signals population decay onto S_0, as states are closest at the conical intersection geometry (see Fig. 9), where the orbital energies are close to degenerate. We notice that the initial binding energy for $S_1 \to D_0$ is predicted at 2 eV, lower than the correct value of ≈ 3.5 eV. This comes from both an overestimated high first excitation energy and an underestimated first ionisation energy (cf. Fig. 9).

On the ground state, the only significant contribution is from the $S_0 \to D_0$ channel, as the $S_0 \to D_1$ channel is Koopmans-forbidden (and high energy). The $S_0 \to D_0$ channel shows a quick decay before the emergence of a strong feature at $t = 45$ fs, mainly due to hot NBD formation.

The spectrum of XMS-CASPT2(2,2), shown in Fig. S2, shows qualitatively the same features as CASSCF(2,2) despite the different dynamics. The meeting of the two excited state channels is earlier, reflecting the faster decay (cf. Fig. 1C), and the $S_0 \to D_0$ channel shows a strong signal from mainly vibrationally excited QC ground state dynamics.

For the XMS-CASPT2(4,4) dynamics, shown in Fig. S3, the $S_1 \to D_0$ channel is strongly reminiscent of the two (2,2) methods. The $S_1 \to D_1$ and $S_1 \to D_2$ channels together make up the $S_1 \to D_1$ channel in the (2,2) methods, understood by comparing the potential energy cuts of XMS-CASPT2(2,2) and XMS-CASPT2(4,4) (in Fig. 9, right panel). This difference is just an artefact of the adiabatic labelling, and the signal comes from the transition of $|20u0\rangle$ character (see final column of Table 1). Finally, the $S_1 \to D_3$ channel, which

[1] Unless one measures photoelectron angular distributions, which give information about the angular momentum of the departing free electrons relative to polarisation axis of the probe pulse. In that case, it might be possible to separate the individual channels.

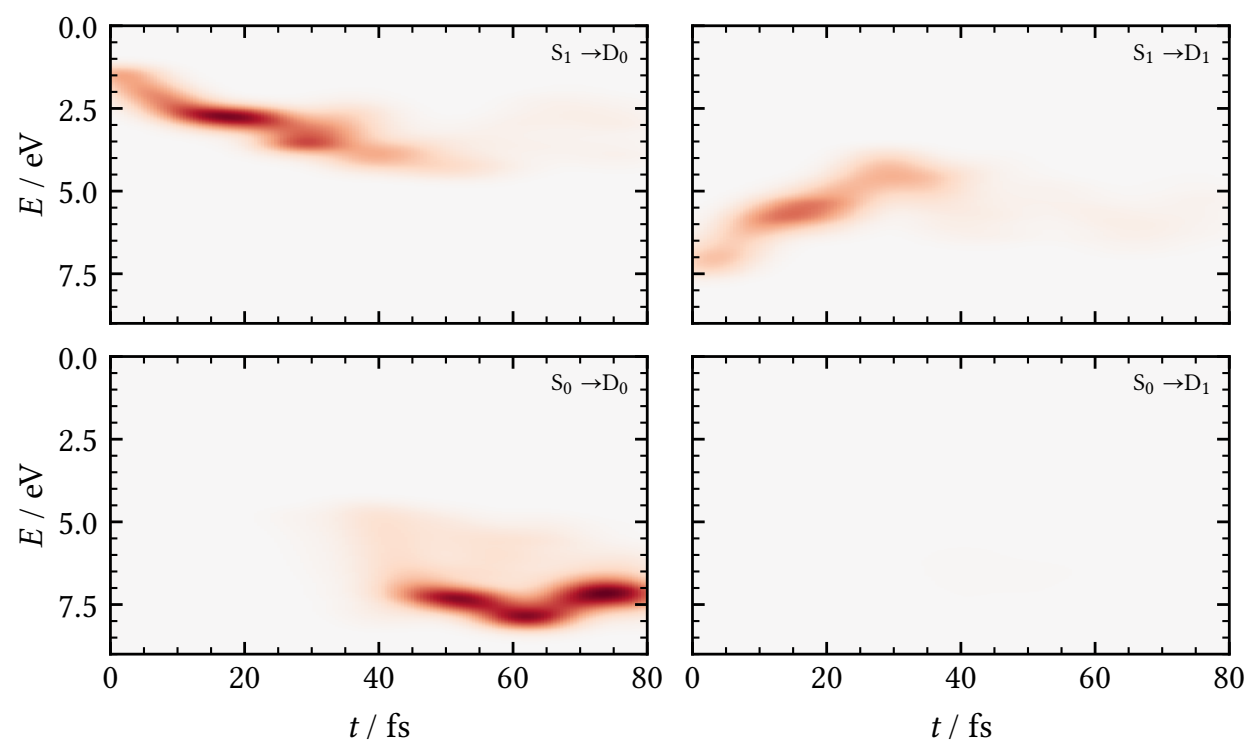

Figure S1: Ionisation channels for CASSCF(2,2) dynamics. Ionic states were calculated using CASSCF(1,2), with cross-sections calculated with the Dyson norm approximation. The $S_1 \to D_0$ descends from $E_{\mathrm{BE}} \approx 2$ eV to $E_{\mathrm{BE}} \approx 4$ eV, meeting with the rising $S_1 \to D_1$ channel at 40 fs. After that, most of the signal is from the $S_0 \to D_0$ ground state channel, which oscillates around the ground state minima, and no $S_0 \to D_1$ signal is seen.

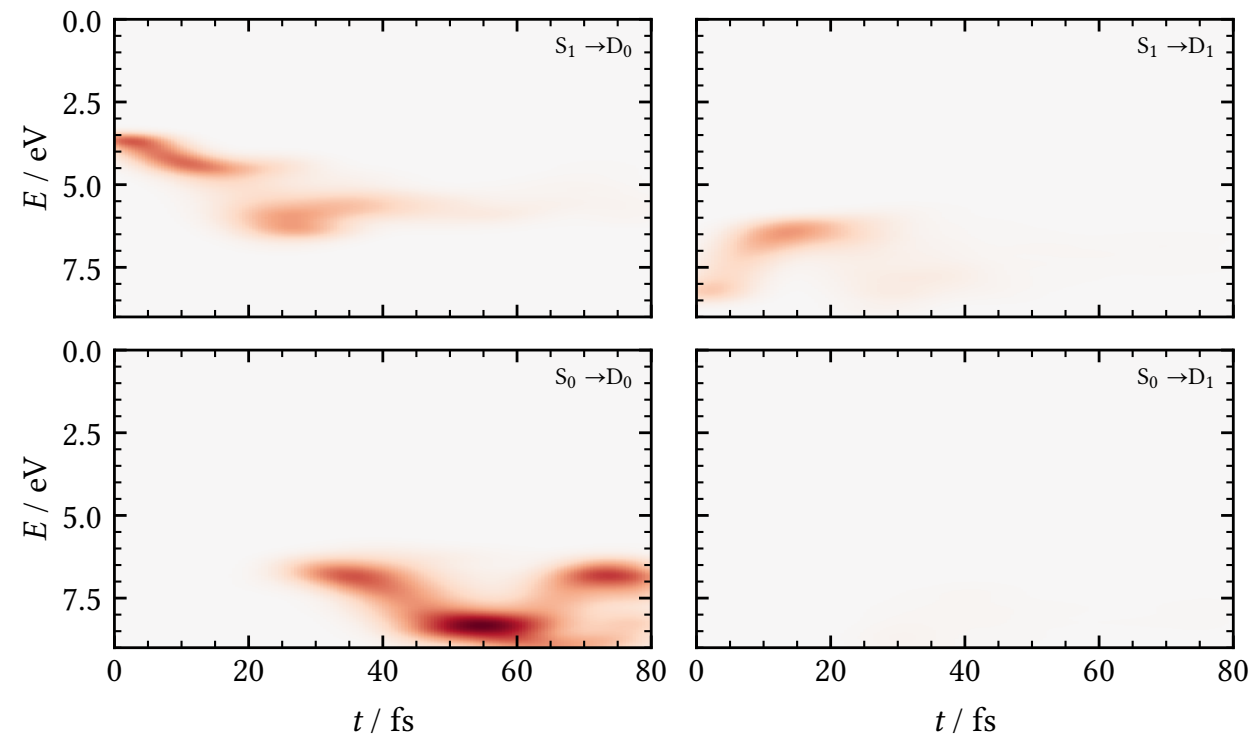

Figure S2: Ionisation channels for XMS-CASPT2(2,2) dynamics. Ionic states were calculated using XMS-CASPT2(1,2), with cross-sections calculated with the Dyson norm approximation. This displays very similar behaviour to the CASSCF(2,2) spectra (Fig. S1), except notably quicker and at higher binding energies.

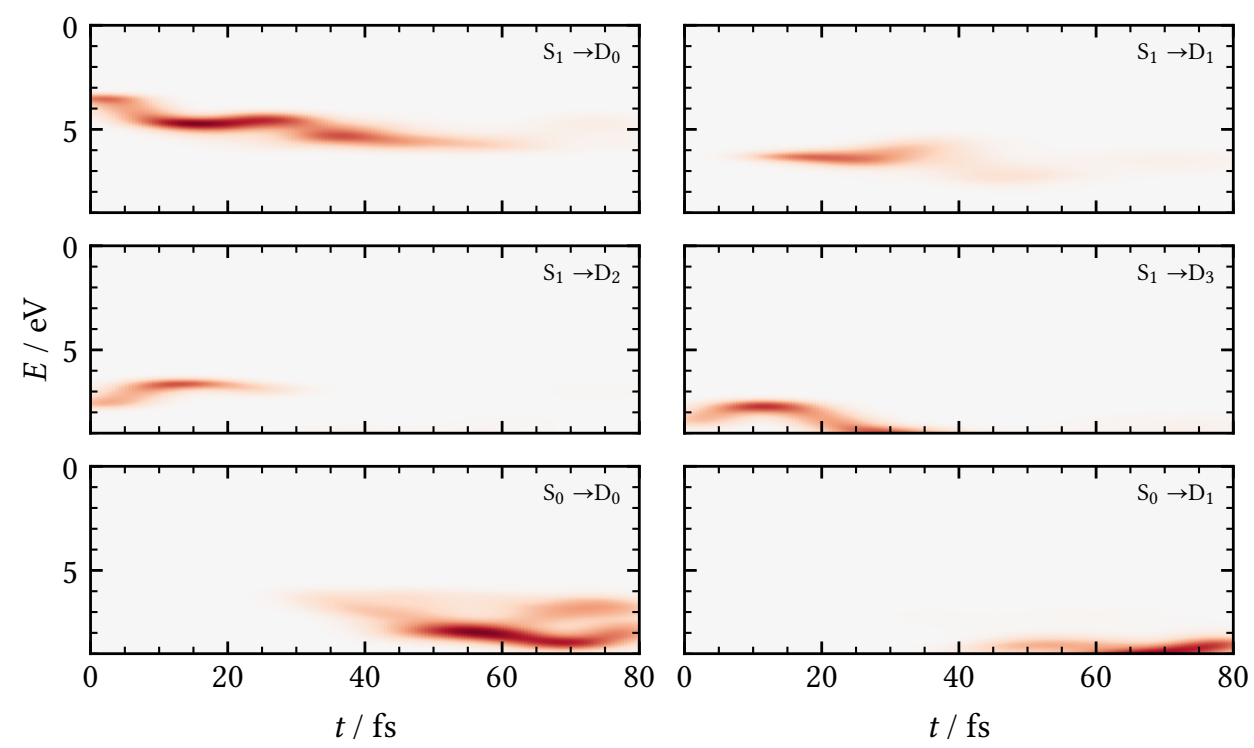

Figure S3: Ionisation channels for XMS-CASPT2(4,4) dynamics. Ionic states were calculated using XMS-CASPT2(3,4), with cross-sections calculated with the Dyson norm approximation.

cannot be calculated in the (2,2) methods, shows a feature at very high binding energy very early in the dynamics, likely obscured by the ground-state bleach.

When the dynamics passes onto the ground state at around 40 fs, the $S_0 \to D_0$ channel shows similar results to the (2,2) active space methods, but we can see the presence of two signals — clear evidence of *both* NBD and QC formation, at least in more equal proportions than in the previous two cases.

2 Binding energies

Table S1: Ionisation energies for the methods, calculated at NBD and QC minimum geometries (taken from Ref. 1). All calculated with the p-cc-(p)VDZ basis. Values marked with a † are from calculations with incorrect (4,4) active spaces (the (4,4) active space is unstable around QC), and are only given to contextualise the results in the main text.

	ΔE / eV	
Method	NBD $S_0 \to D_0$	QC $S_0 \to D_0$
CASSCF(2,2)	7.65	7.16
XMS-CASPT2(2,2)	8.73	8.25
XMS-CASPT2(4,4)	8.42	7.90†
CASSCF(4,4)	8.34	7.22†

References

1. J. C. Cooper & A. Kirrander, *Phys. Chem. Chem. Phys.*, **2025**, 27, 3089-3101
